# Bacterial colonization dynamics and antibiotic resistance gene dissemination in the hospital environment after first patient occupancy: a longitudinal metagenetic study

**DOI:** 10.1186/s40168-021-01109-7

**Published:** 2021-08-11

**Authors:** Tilman E. Klassert, Rasmus Leistner, Cristina Zubiria-Barrera, Magdalena Stock, Mercedes López, Robert Neubert, Dominik Driesch, Petra Gastmeier, Hortense Slevogt

**Affiliations:** 1 Jena University Hospital, ZIK Septomics, Host Septomics, Jena, Germany; 2 Institute for Hygiene and Environmental Medicine and Department for Medicine (Gastroenterology, Infectious diseases, Rheumatology), Charité – Universitätsmedizin Berlin, Berlin, Germany; 3 University Institute of Tropical Diseases and Public Health of the Canary Islands, University of La Laguna, San Cristóbal de La Laguna, Spain; 4 BioControl Jena GmbH, Jena, Germany; 5 Institute for Hygiene and Environmental Medicine, Charité–Universitätsmedizin, Berlin, Germany

## Abstract

**Background:**

Humans spend the bulk of their time in indoor environments. This space is shared with an indoor ecosystem of microorganisms, which are in continuous exchange with the human inhabitants. In the particular case of hospitals, the environmental microorganisms may influence patient recovery and outcome. An understanding of the bacterial community structure in the hospital environment is pivotal for the prevention of hospital-acquired infections and the dissemination of antibiotic resistance genes. In this study, we performed a longitudinal metagenetic approach in a newly opened ward at the Charité Hospital (Berlin) to characterize the dynamics of the bacterial colonization process in the hospital environment after first patient occupancy.

**Results:**

The sequencing data showed a site-specific taxonomic succession, which led to stable community structures after only a few weeks. This data was further supported by network analysis and beta-diversity metrics. Furthermore, the fast colonization process was characterized by a significant increase of the bacterial biomass and its alpha-diversity. The compositional dynamics could be linked to the exchange with the patient microbiota. Over a time course of 30 weeks, we did not detect a rise of pathogenic bacteria in the hospital environment, but a significant increase of antibiotic resistance determinants on the hospital floor.

**Conclusions:**

The results presented in this study provide new insights into different aspects of the environmental microbiome in the clinical setting, and will help to adopt infection control strategies in hospitals and health care-related buildings.

**Video Abstract**

**Supplementary Information:**

The online version contains supplementary material available at 10.1186/s40168-021-01109-7.

## Background

As modern humans, we spend up to 90% of our time in indoor environments [1]. Microorganisms that inhabit the same indoor environments constitute an ecosystem that is in continuous exchange with us. This exchange of microbes is pivotal for the microbial assemblages and community structures in built habitats [2]. From an anthropocentric point of view, the environmental exposure to microorganisms will conversely also impact the human microbiome patterns and, consequently, the health of the inhabitants [1, 3, 4]. In the last decades, the scientific community has begun to investigate the microbial interactions between humans and their built environment by characterizing the microbial diversity and ecology of a large number of constructed habitats. These included, among others, residences [5], museums [6], office buildings [7], public restrooms [8], subways [9, 10], and hospitals [11–13].

Among the built environments which have been screened for their microbial composition, hospitals and healthcare centers might have the most immediate effect on human health. The environmental microorganisms may directly influence patient recovery and outcome. Thus, the clinical environment is subjected to stringent hygiene guidelines [14]. The hygienic standards in hospitals include several sterilization-, disinfection-, and antisepsis-measures [15]. In addition, different architectural strategies are taken into account as potential modulators of the environmental microbiome in healthcare buildings [16–19]. Despite the scientific and technological advances, hospital-acquired infections (HAIs) remain a major threat and one of the top public health issues worldwide [20]. This problem is further aggravated by the rise of infections involving antibiotic-resistant bacteria [21, 22]. An understanding of the bacterial community structure in the hospital environment may help to develop new approaches to reduce the spread of nosocomial pathogens and the dissemination of antibiotic resistance determinants.

Until recently, culture-based microbiology methods were the first and only available option to monitor hygiene standards or to track specific HAIs in the clinical setting [23]. Metagenomics and next-generation sequencing (NGS)-based metagenetic approaches have opened an avenue for the comprehensive characterization of the total microbial diversity in a culture-independent manner [24]. Moreover, they allow for the parallel detection of additional microbiologic aspects such as virulence or resistance determinants in complex communities [24, 25]. However, only a few studies have addressed the environmental microbiome of the hospital in such a comprehensive manner [11, 26, 27]. Most of the environmental studies investigate the microbial profiles of intensive care units (ICUs), especially in the neonatology [28–31]. Normal hospital wards represent potentially different hospital environments, providing additional ecological conditions for patient-microbe interactions that could have important clinical implications. Recent studies and data from surveillance programs for nosocomial infections report a high incidence of HAIs in hospital wards, such as neurology stations [32, 33]. However, hospital wards have been addressed in only a few number of microbiome studies, investigating different room sites [11, 12]. Moreover, the colonization dynamics, and specifically the kinetics of the microbial succession after hospital opening and first patient occupancy are still poorly understood.

In this study, metagenetic approaches were used to characterize the compositional changes of the colonizing bacterial communities in the environment of a neurology ward at the newly constructed Charité Hospital bed tower in Berlin. The longitudinal study addresses the pre-opening microbiome and its compositional progression over the first 30 weeks after patient occupancy. Beyond community structures, the study investigates the dissemination of antibiotic resistance genes (ARGs) on floors, doorhandles and sinks, as part of the resistance-reservoir observed in the clinical setting.

## Material and methods

### Study design and sample collection

This study was designed to investigate the bacterial colonization dynamics on the environmental elements of a newly opened neurology ward at the Charité–Universitätsmedizin Berlin. The survey timeline covered the pre-opening week and the first 30 weeks following patient occupancy. Sampling was performed on a weekly basis, and included 3 different environmental sites of the patients’ rooms: the floor, the doorhandle, and the sink. These 3 sites were selected after a pilot study for yielding a relative high biomass (as compared to other sites such as wall and handrail) and for showing the highest diversity coverage of the environmental microbiota in our particular setting. The sampled sites were cleaned daily using site-specific disinfectants (ECOLAB, Germany, see Suppl. Fig. S1 for disinfectant composition and sampling details). The sampling was performed at least 2 h after cleaning and covered 9 independent rooms of the neurology ward (Stations 116A/B). Patient samples (nose swab, rectal swab) were collected from these rooms during the whole timespan (room occupation rate: 91.7%; patient parameters in Suppl. Table S1). In addition, elbow- and handpalm-swabs (right side) were collected from the patients during the final 6 weeks. All patients gave written informed consent in accordance with the Declaration of Helsinki and the local ethics committee. Environmental temperature and humidity data were recorded for each of the rooms during the weekly sampling routine using a Thermo-Hygrometer BC25 (Trotec, Germany).

A total of 1547 samples (including 854 environmental samples, 408 patient samples, and 285 blank controls) were processed throughout the course of this study. Environmental samples and patient material were sampled by trained study-nurses. In all cases, sterile swabs were premoistened with saline solution and rubbed at the collection site. The swab was then resuspended in 200 μl TE buffer, and immediately frozen at − 80 °C until further processing. The blank controls consisted of saline solution used for the premoistening of the swabs in each of the rooms and were processed following identical protocols.

### DNA extraction and *16S rRNA* gene quantification

The DNA of all samples was extracted with the innuPREP Bacteria DNA kit (Analytik Jena, Germany) following manufacturer’s instructions. The bacterial biomass in the samples was then measured by quantification of the *16S rRNA* gene copies using a qPCR approach. In brief, 4 μl of DNA were used as template in a 20-μl SYBR-Green-based qPCR reaction (BioLine, UK) with 200 nM specific amplification primers for the V4 region of the *16S rRNA* gene (515Fw: 5′-GTGYCAGCMGCCGCGGTAA-3′; 806Rv: 5′-GGACTACNVGGGTWTCTAAT-3′). The qPCR reactions were set up in a CAS-1200 pipetting robot (Qiagen, Netherlands) and run in technical duplicates on a Rotor-Gene Q cycler (QIAGEN, Netherlands). The cycling conditions included an initial denaturation step (95 °C, 10 min) and 40 amplification cycles (95 °C, 15 s; 58 °C, 20 s; 72 °C, 30 s), followed by a melting curve to assess the specificity of the amplification process. Non-template controls were included in each run to control for potential contamination. Quantification of absolute target copy numbers was performed using the standard curve method (*R*^2^ = 0.996; conc = 10^(− 0.246*CT + 10.177)) as implemented in the Rotor Gene Series software v. 2.1.0 (QIAGEN, Netherlands).

### Library construction and sequencing

*16S rRNA* amplicon sequencing was used for bacterial profiling of all environmental and patients’ samples. The library construction was performed as described elsewhere [34, 35]. In brief, the amplification primers 515Fw/806Rv were fused with Golay barcodes and adapter sequences (see details in Suppl. Table S2). These constructs were then used as primers to generate the library by PCR. The 50 μl reaction was performed on a S1000 Thermal Cycler (BioRad, USA) using the Platinum PCR SuperMix (Thermo Fisher Scientific, USA). Non-template controls were used to control potential contamination during the amplification process. Thermal conditions included an initial denaturation step (94 °C, 3 min), followed by 35 amplification cycles (94 °C, 15 s; 58 °C, 20 s; 72 °C, 30 s) and a final elongation step at 72 °C for 10 min. PCR products were purified by size-selection on 2% SizeSelect E-Gels (Thermo Fisher Scientific, USA) and quantified on D1000 Tapes using a TapeStation 2200 (Agilent Technologies, UK). The libraries were equimolarly pooled and prepared for Illumina sequencing using the MiSeq Reagent Kit v2 (Illumina) and following manufacturer’s instructions. Run plan and sequencing reagents and primers were adapted according to Caporaso et al. [36]. Sequencing was performed on a MiSeq apparatus (Illumina) with 251 cycles.

### Sequencing data analyses

Fastq files were first quality checked using FastQC [37]. Forward and reverse reads were then quality-trimmed using Trimmomatic [38] and demultiplexed with QIIME v1.9.1 scripts [39]. A sequence-based filtering method was applied to remove potential contaminants from the sequencing data as described elsewhere [40]. The detailed pipeline is available at GitHub (https://github.com/ZubBar/Sequence-based-filtering-method-for-16S-rRNA-sequencing.git). To determine the bacterial taxonomic distribution in the samples, reads were first clustered using the open reference OTU picking method with a sequence identity cutoff of 97% (implemented in the QIIME pipeline) followed by the taxonomic assignment using the SILVA REF NR 99 (release 132) database [41]. OTUs represented at less than 0.2% relative abundance were not shown. Taxonomic classification at species level was performed using the sub-classifying genus option implemented in SILVA, in order to avoid loss of data when species level annotation was not possible. Sub-classified genus results were listed as “sp.” annotations. Principal coordinate analyses (PCoA) were calculated using weighted or unweighted UniFrac distances. Statistical significance of beta-diversity metrics between groups was assessed by analyses of similarities (ANOSIM). The alpha-diversity metrics were performed using Shannon indices. To identify the core microbiota (OTUs in at least 50% of the samples), the compute_core_microbiome.py scripts of the QIIME software was used. The datasets generated in this study (trimmed, demultiplexed, and sequence-based filtered FASTA-files) are available at the SRA database under the accession number: PRJNA672813 [https://www.ncbi.nlm.nih.gov/sra/PRJNA672813]. Further project metadata, biom-tables and diversity metrics can be found at the Zenodo platform [DOI: 10.5281/zenodo.4600715].

### Network analyses

In order to measure non-random interactions between bacteria, co-occurrence network analyses were calculated with the SparCC software v.0.1.0 [42] using an OTU table with limited sequences presented at a relative abundance > 0.3%. The pairwise median correlation was estimated using twenty interactions, and the statistical significance of each correlation was calculated by bootstrapping (with 100 interactions). All statistically significant (*p* < 0.05) SparCC correlations with a magnitude > 0.9 were incorporated into the network analyses. Each OTU of the reconstructed networks was represented as a node, and the significant correlations between the nodes depicted as edges. The network structure was further supplemented with the clustering coefficient values and the modularity indices, as additional measure of structural stability of the communities [43]. The networks were visualized using the interactive platform Gephi [44], and the nodes defined and colored based on their pathogenicity (Suppl. Table S3).

### Statistics

Pairwise comparisons between groups were performed using student *t* tests or one-way ANOVA with a significance threshold of *p* < 0.05. Statistical analyses and graphic presentations were performed using GraphPad Prism 5.0 (GraphPad Software, USA). Linear regression models were employed to investigate trend changes across multiple timepoints/datasets, in order to detect a significant increase or decrease of the bacterial biomass. The Bonferroni method was used for multiple testing corrections.

### Antibiotic resistance gene detection

The presence of 12 antibiotic resistance genes (ARGs) (see Suppl. Table S4) conferring resistance to beta-lactams, quinolone, polymyxin, and methicillin was analyzed throughout the course of the study. Following determinants were addressed: *blaKPC*, *blaNDM*, *blaOXA48*, *blaVIM*, *blaCMY*, *blaGES*, *blaSHV*, *blaTEM*, *blaCTX-M1*, *qnrB1*, *mcr1*, and *mecA*. These include many of the most relevant ARGs [45–51] isolated from nosocomial pathogens, as reported in European surveillance programs [52]. Furthermore, most of these genes have been reported to be potentially transferrable between different bacteria by horizontal gene transfer [46, 53]. For their detection, custom multiplex Taq-Man assays were developed as described previously [54, 55]. The real-time qPCRs were carried out using the RNA UltraSense One-Step Quantitative RT-PCR System Kit (Thermo Fisher Scientific, USA). The qPCR reactions, containing 200 nM of each primer and 320 nM of the respective Taq-Man probes (see sequences in Suppl. Table S4), were set up in a CAS-1200 pipetting robot (QIAGEN, Netherlands) and run in technical duplicates on a Corbett Rotor-Gene 6000 cycler (QIAGEN, Netherlands). Thermal conditions included an initial 95 °C denaturation step for 4 min, followed by 40 cycles of denaturation at 95 °C for 30 s, initial annealing at 50 °C for 30 s (60 °C after cycle 3), and extension at 72 °C for 60 s. The data were collected during the annealing phase and analyzed using the Rotor-Gene 6000 software v.2.1.0 (QIAGEN, Netherlands). DNA samples of bacterial colonies positive for the different ARGs were used as controls in each multiplex PCR. Samples were considered as positive for a specific ARG when the achieved cycle threshold (Ct) was < 35.

## Results

### Bacterial colonization dynamics of the hospital environment

**Fig. 1:**
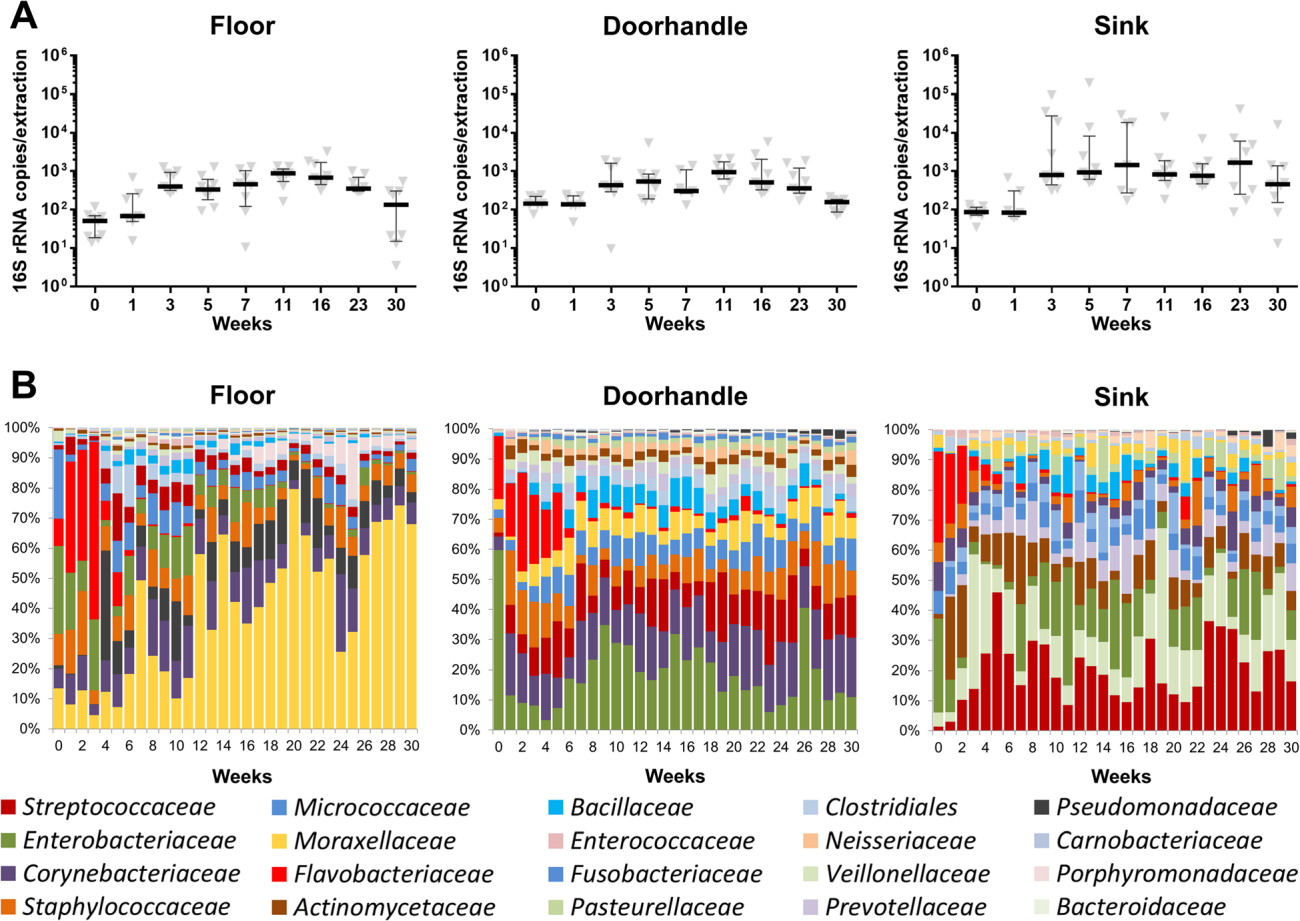
Bacterial colonization dynamics of the floor, the doorhandle and the sink during the first 30 weeks after patient occupancy. **A** Quantitative analysis of the bacterial biomass over time as measured by qPCR. Shown are the *16S rRNA* gene copies in each sample (Median ± IQR). **B** Taxonomic summary of the compositional changes of each environmental site over time. Shown are the relative abundances of the collapsed main taxa (> 0.5%) at family level for each week

Genetic approaches were used to investigate quantitative and compositional aspects of the bacterial colonization patterns of the hospital environment. Absolute quantification of the *16S rRNA* copies showed an increase of the microbial biomass during the first weeks after patient occupancy. For all three tested sites (floor, doorhandle and sink), this increase was significant (*p* < 0.05, linear regression) over the first 7 weeks (Fig. 1A). After a short stabilization phase, the biomass measurements showed a slight decrease of the bacterial loads toward the end of the sampling period (between weeks 16 and 30), reaching statistical significance (*p* < 0.05, linear regression) for both the floor and the doorhandle samples. This reduction of bacterial load correlated with seasonal changes of indoor temperature and relative humidity at sampling time as shown by multiple regression models including these two physical factors (Suppl. Fig. S2).

The sequencing analysis of the 854 environmental samples showed microbial community patterns with rapid taxonomic successions over time. The compositional changes were highly site-specific and most prominent during the first 5–7 weeks after patient occupancy. So, the pre-opening floor was dominated by *Enterobacteriaceae* (21.28%) and *Micrococcaceae* (16.70%). After patient occupancy, the floor microbiota was steadily colonized by *Moraxellaceae* over time, while experiencing a reduction in *Enterobaacteriaceae* and *Flavobacteriaceae* (Fig. 1B). The doorhandle samples were characterized by *Enterobacteriaceae* (58.15%) and *Flavobacteriaceae* (20.31%) in the week before hospital opening. After the initial week of patient occupancy, the relative abundance of *Enterobacteriaceae* decreased (5.5-fold decrease) while *Corynebacteriaceae* increased in a significant manner (4.3-fold increase). For *Flavobacteriaceae*, we observed a steady reduction across the 6 initial opening weeks, after which the presence of this family was permanently < 4% (Fig. 1B). On the other hand, the sink samples were mainly colonized by *Veilloneaceae* and *Streptococcaceae* during the first few weeks of patient occupancy (Fig. 1B).

**Fig. 2:**
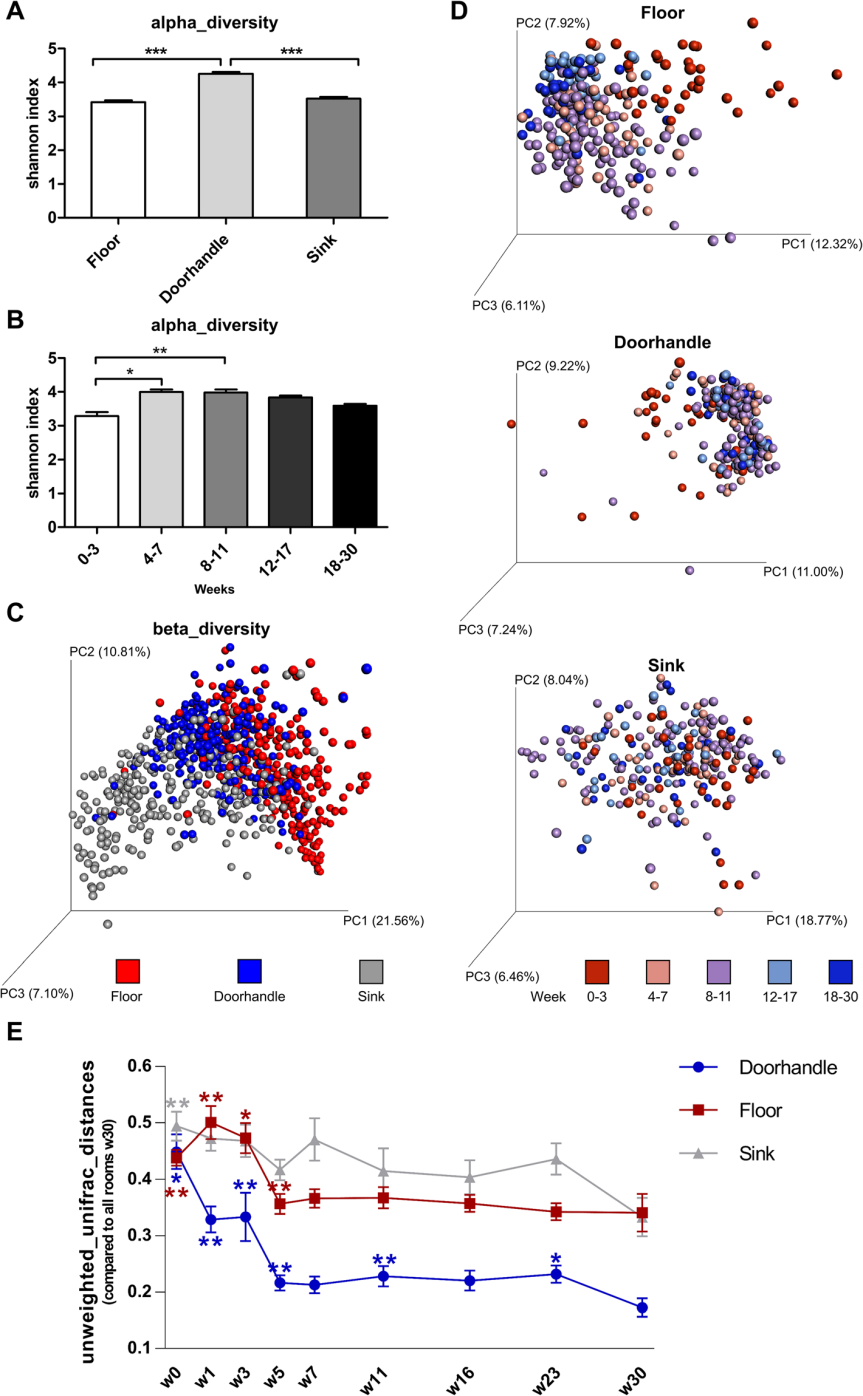
Diversity metrics of the environmental samples between sites and over time. **A** Alpha-diversity of each environmental site. Shown is the Shannon index (Mean ± SEM, ***p < 0.001, ANOVA/Bonferroni). **B** Alpha-diversity over time. Shown is the Shannon index over different week blocks (*p < 0.05, **p < 0.01, ANOVA/Bonferroni). **C** Principal coordinates analysis of the beta-diversity using weighted UniFrac distances. Shown are the distances between the different sites addressed in this study. **D** Principal coordinates analysis showing the unweighted UniFrac distances between samples for each site as analyzed over time in different week-blocks. **E** Graph depicting the stabilization dynamics of the bacterial community structures as measured by the unweighted UniFrac distances to the microbial composition of all rooms in week 30 (Mean ± 95%CI). Statistical significance was calculated by ANOSIM between each week and week 30. (*p < 0.05, **p < 0.01, after Bonferroni correction for multiple testing)

Diversity metrics showed the highest alpha-diversity for the doorhandle samples, which was significantly increased over the other two environmental sites (Fig. 2A). In order to analyze the microbiota changes over time, the weeks were grouped in different week blocks based on the alpha- and beta-diversity metrics and the distance pattern between consecutive weeks (Suppl. Fig. S3). The overall alpha-diversity of the environmental microbiota increased in a significant manner when comparing the first week block (w0-3) with the two following (w4-7 and w8-11; see Fig. 2B). When analyzing the sites independently, this diversity increase was most prominent in the floor samples (Suppl. Fig. S4).

The beta-diversity measurements showed a clear site-specific pattern as reflected by the spatial distance between the clusters generated in a PCoA plot (Fig. 2C). Beta-diversity analysis of each site revealed a fast colonization process, as we observe an early segregation of the week blocks toward a definite community structure (Fig. 2D). In all three sites, early week blocks (w4-7 or w8-11) already clustered with late week blocks as they approach their stable configuration in the PCoA space. This conclusion was further supported by the measures of distance to the final microbial composition (last sampling week). The unweighted UniFrac distances to the week-30-microbiome decreased in all sites over the first few weeks (w1-w5). As early as in week 7, we observed no statistical difference in the composition of the microbial communities when compared to the last sampling week (w30), as assessed by pairwise ANOSIM of the beta-diversity. These data suggest that the environmental microbiome of a newly constructed hospital might be stably established after only a few weeks of patient occupancy. Furthermore, the site-specific aspect of the microbial colonization process was reinforced by the observation of growing distances between the different bacterial communities over time (Suppl. Fig. S5). Moreover, the microbiome maturation was consistent in all rooms, and no significant spatial effects such as distance between rooms could be detected (Suppl. Fig. S6).

### Impact of patient occupancy on the bacterial colonization patterns of the environment

In a next step, we determined the extent of the microbial exchange and interaction between environmental- and patient-sites analyzed in each of the rooms. In total, we analyzed 408 patient samples over the time period of 30 weeks after hospital opening. The taxonomic characterization of the nasal and rectal swabs allowed us to identify a distinct microbiome pattern for each site (Suppl. Fig. S7A). Barring inter-individual differences and outliers, the nare samples were mainly dominated by *Corynebacteriaceae* and *Staphylococcaceae*, while the rectum samples were rather characterized by the presence of *Clostridiales* and *Prevotellaceae*. In order to measure the exchange between the patient material and the environmental samples, we analyzed the overlap of the core microbiomes of each site across different week blocks. The amount of shared taxa between all sites increases over time, especially during the initial weeks (from week block w0-3 to week block w4-7; Suppl. Fig S7B). These results suggest an important impact of the patients on the establishment of the environmental microbiome in this early time frame. In comparison, other external factors, such as temperature or humidity, showed a very small impact on the colonization dynamics (Suppl. Fig. S8).

**Fig. 3:**
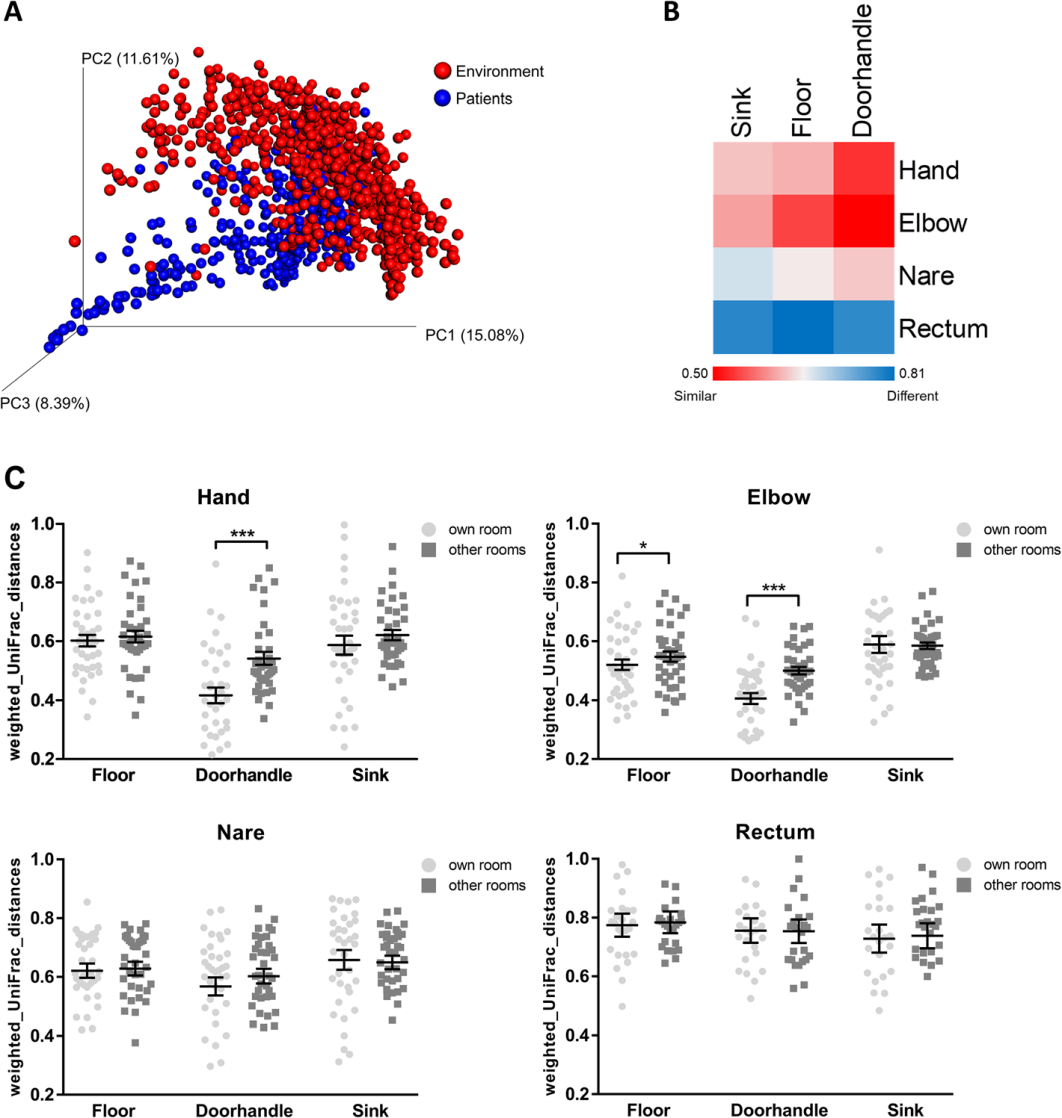
Effect of patient occupancy on the bacterial colonization patterns. **A** PCoA of the beta-diversity depicting the weighted UniFrac distances between environmental and patient samples. **B** Heatmap showing the similarity scores between sample pairs after establishment of the environmental microbiome (final 6 weeks). **C** Comparative analysis of the pairwise weighted UniFrac distances between patients in single-rooms and the environmental sites from either the own occupied room or the other rooms analyzed in the study (mean distance to all rooms). Each dot represents the distance between a patient site and an environmental site in a specific week. (*p < 0.05, ***p < 0.001, paired *t* test)

During the last 6 weeks of sampling, two additional skin sites (handpalm and elbow) of the patients were included in the study to determine the extent of their microbial interchange with the final environment community. Hand and elbow samples were both dominated by *Corynebacteriaceae* and *Staphylococcaceae*, two commensal taxa of the human skin (Suppl. Fig. S7C). When the environmental samples of all 3 room sites were plotted together with the samples from the 4 patient sites on a PCoA space, we observed a significant segregation of the patient- and the environmental-cluster (*p* < 0.001, ANOSIM; Fig. 3A). To determine the differential interaction between sites, the weighted UniFrac distances were compared pairwise and plotted as heatmap (Fig. 3B). These comparisons identified the skin samples (hand and elbow) as most related to the environmental samples, and especially similar to the doorhandle samples. In contrast, the rectal samples showed the highest distance to the environmental sites (see also Suppl. Fig. S9).

Furthermore, the beta-diversity metrics revealed a site-specific effect of each particular patient on the composition of the bacterial communities of their specific room. The individual impact during the short period of hospitalization (mean length of stay (LOS) = 5.8 days) was significant for the skin samples (hand and elbow), as shown by the pairwise comparison of distances between particular patient sites and environmental sites from either their own occupied room or the other rooms addressed in this study (Fig. 3C). These data suggest that the skin microbiome dictates the immediate impact of a particular patient on the environmental microbiota, and that this effect can be measured as early as a few days after admission.

### Taxonomic succession and community structure over time

As already shown, a significant quantitative and compositional shift was observed after patient occupancy in all three analyzed room sites. The *Flavobacteriaceae*-dominated environment steadily evolved toward stable microbial communities with a site-specific structure. After hospital opening, the floor was characterized by the increase of *Moraxellaceae*, the doorhandle was colonized by *Corynebacteriaceae* and *Streptococcaceae*, and the sink was dominated by *Streptococcaceae* and *Veillonellaceae*, among others (see also Suppl. Fig. S10). Analyses at deeper taxonomic levels (species-level) targeting the significant changes in taxa abundance between sites showed the extent of the site-specificity during this early colonization process. The Kruskal-Wallis test identified 42 OTUs as significantly changed between the three environmental sites, the top 10 of which are listed in Table 1.

**Table 1:** Differentially abundant OTUs in each environmental site. Shown are the top 10 significant OTUs after Kruskal-Wallis test with Bonferroni correction and the mean counts at each of the sampled room sites

Species	Bonferroni_P	Floor	Doorhandle	Sink
*Pseudomonas* sp.	1.96E-77	720.21	15.68	29.71
*Acinetobacter* sp.	7.76E-69	3572.17	197.35	174.29
*Rheinheimera* sp.	6.15E-67	508.66	8.30	15.00
*Micrococcus* sp.	5.65E-52	157.35	118.02	33.82
*Lawsonella clevelandensis*	9.74E-48	23.51	174.19	25.96
*Corynebacterium kroppenstedtii*	2.38E-40	21.39	223.22	21.69
*Corynebacterium ureicelerivorans*	2.90E-35	206.48	80.02	37.73
*Corynebacterium testudinoris*	1.25E-29	185.11	51.55	67.15
*Staphylococcus aureus*	5.38E-29	509.94	281.90	127.88
*Corynebacterium glyciniphilum*	4.53E-26	305.90	114.03	80.43

**Fig. 4:**
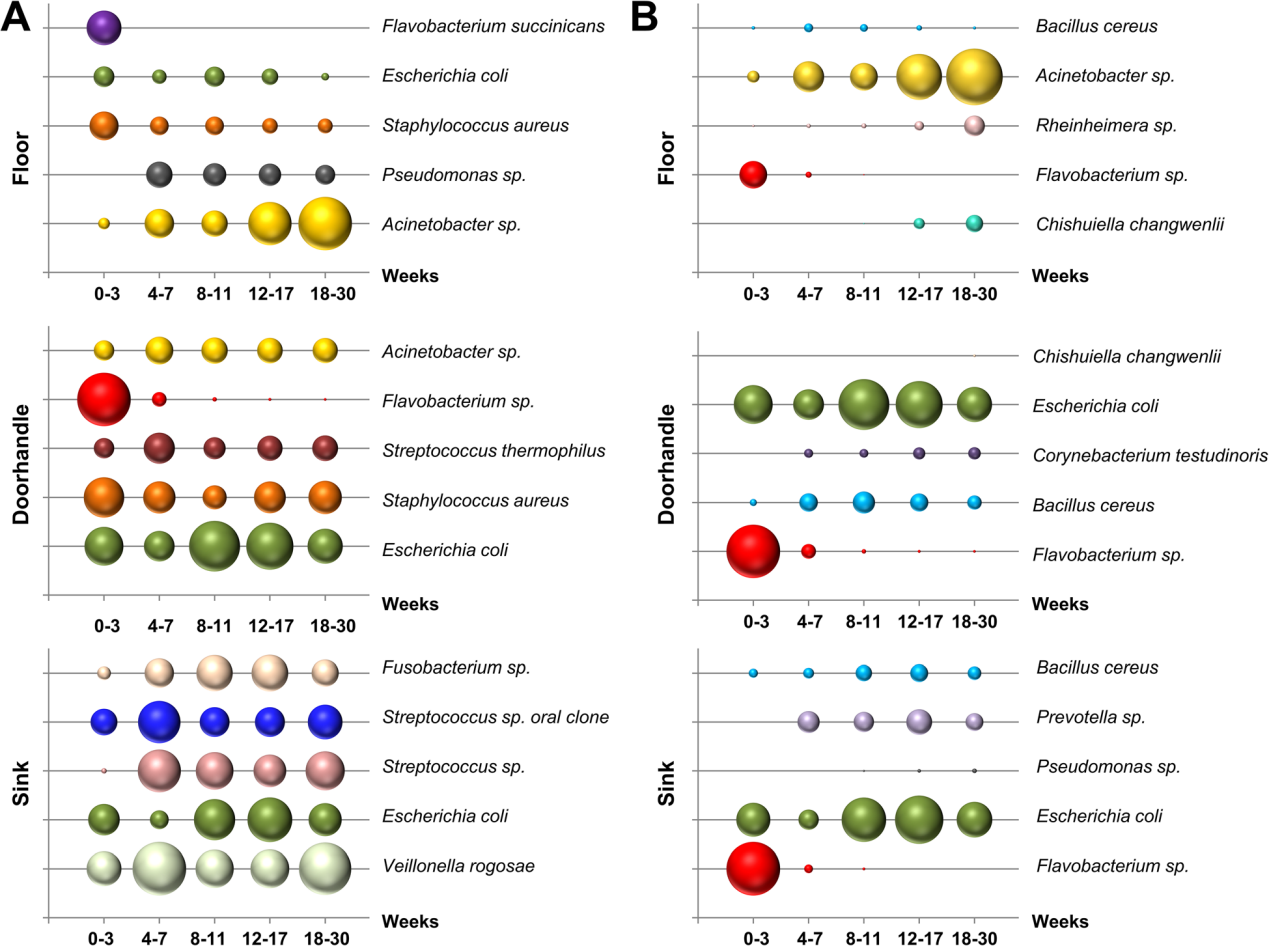
Dynamics of the bacterial community structure over time. **A** Relative abundance changes over time of the most abundant species. Bubble size represents the proportional abundance across different week blocks. **B** Relative abundance dynamics of the top significantly changed taxa (species level) across the different week blocks

When analyzing relative species abundance by sites, *Acinetobacter* sp. and *Pseudomonas* sp. were identified as the most prominent taxa at the later stages of the microbial colonization of the hospital floor. *Escherichia coli* and *Staphylococcus aureus* were the most abundant species in the doorhandle samples, and *Veillonella rogosae* and *Streptococcus* sp. dominated the sink samples (Fig. 4A). In a next step, the taxa were arranged according to their variation over time, as measured by statistical significance after Kruskal-Wallis-testing. Figure 4B depicts the fluctuation of the relative abundance over time for the top 5 taxa that were significantly changed (Fig. 4B). In many cases, the most abundant taxa of each site were also among the significantly changed ones. So, among others, the most important pattern changes were observed for *Acinetobacter* sp. on the floor, *E*. *coli* and *Bacillus cereus* at the doorhandle, and *Prevotella* sp. and *B*. *cereus* in the sink. In all three sites, we also observed the significant decrease of *Flavobacterium* sp. A directed search for pathogenic bacteria in the hospital environment showed that only 3 of eleven common hospital pathogens were detected in any of the environmental samples: *E*. *coli*, *S*. *aureus*, and *Enterococcus faecalis*. However, we could not detect a significant increase in the relative abundance of these pathogens in the room samples after patient occupancy (Suppl. Fig. S11).

**Fig. 5:**
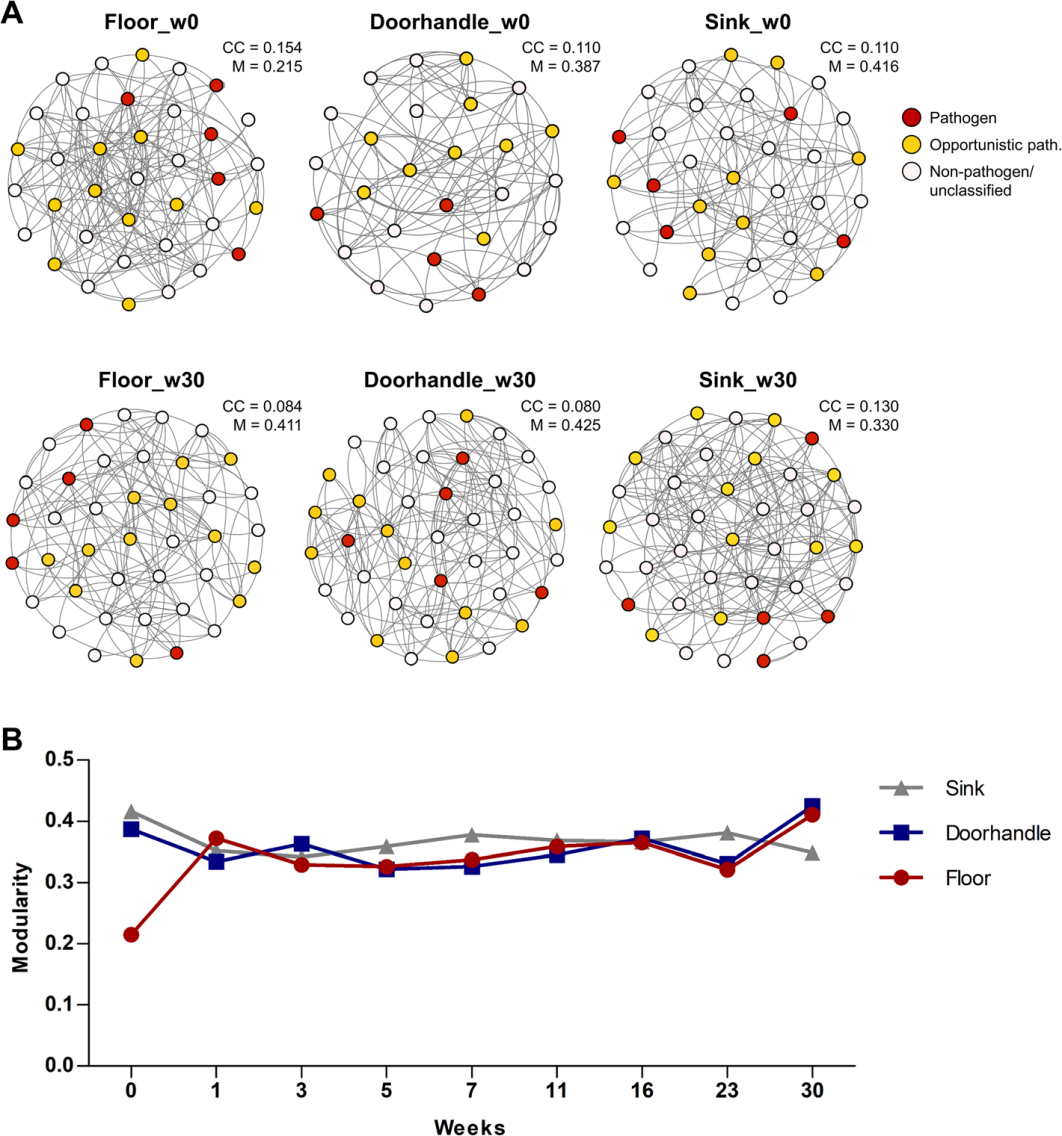
Network analyses of the hospital microbiome in the pre-opening week (w0) and the last sampling week (w30) after patient occupancy. **A** Shown is the connectedness between nodes (colored according to their pathogenic status, see Suppl. Table S3). The clustering coefficient (CC) and the modularity index (M) are depicted for each of the networks. **B** Modularity measured for each room site over time. Shown are the modularity indices of each network as measured in different weeks

The environmental community composition was then analyzed for bacteria interactions using taxonomic units counts extracted from the sequence data. Interaction networks were inferred for the pre-opening week (w0) and for the last sampling week after patient occupancy (w30), as representative for an incipient and a stabilized microbial community structure, respectively. The resulting networks in the pre-opening week (w0) consisted of 36, 27, and 33 nodes in the floor, doorhandle and sink samples, respectively. In all cases, non-pathogenic bacteria were dominant, while the overall clustering coefficients were between 0.11 and 0.15, suggesting a low connectedness of the community members in all three sites. After 30 weeks of patient occupancy, we observed an increase of the node counts, with 40–41 bacteria in each of the interaction networks. The connectedness remained low, with clustering coefficients ranging between 0.08 and 0.13. However, modularity metrics in doorhandle and sink networks were elevated throughout the whole study duration. Interestingly, the floor showed a low modularity in the pre-opening week (*M* = 0.215 in w0), which almost doubled after patient occupancy (*M* = 0.411 in w30), suggesting that the modifications of the community led to an interaction network with a modular structure. A closer look at the kinetics of this modularity change revealed an early onset (first week of patient occupancy) of this structure development (Fig. 5B). Taking all together and in spite of a low overall connectedness between the community members of the environmental microbiota, we observe an increase of the interactions and the node counts for the networks over time, and dense connections within certain groups of bacteria (high modularity).

### Antibiotic resistance gene dissemination in the hospital environment

**Fig. 6:**
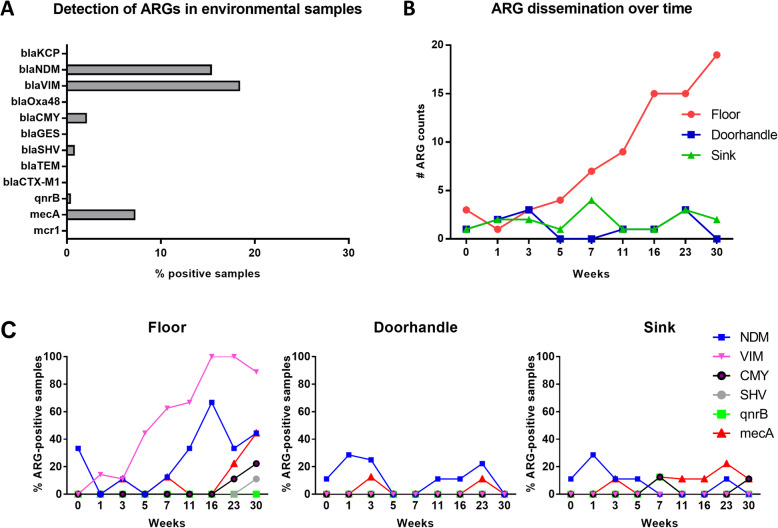
ARG detection in the hospital environment after patient occupancy. **A** Bar chart depicting the ARG expression across all environmental samples analyzed. Bars represent percentage of samples with positive ARG detection. **B** ARG detection over time. Shown are the total ARG counts for each of the environmental sites over different weeks after hospital opening. **C** Chart depicting the specific ARGs detected at each site over time. Shown is the percentage of positive samples

In a next set of experiments, we aimed to investigate whether and to which extent the site-specific development of the environmental microbiome might be associated with the dissemination of antibiotic resistance determinants after hospital opening. Therefore, selected weeks across the 30-week timeline were analyzed for the presence of 12 ARGs conferring resistance to beta-lactams (*blaKPC*, *blaNDM*, *blaOXA48*, *blaVIM*, *blaCMY*, *blaGES*, *blaSHV*, *blaTEM*, *blaCTX*-*M1*), quinolone (*qnrB1*), polymyxin (*mcr1*), and methicillin (*mecA*) using specific real-time qPCR assays (Suppl. Table S4). While half of the tested ARGs were not detected in any of the environmental samples, six ARGs were tested positive in the room samples after patient occupancy (Fig. 6A). The ARGs found to be present in the environmental microbiome included the genes coding for quinolone resistance proteins (*qnrB1*), beta-lactamases (*blaSHV*, *blaCMY*, *blaNDM*, and *blaVIM*) and the penicillin-binding protein PBP2a (*mecA*). Two of the genes conferring resistance to beta-lactams, *blaVIM* and *blaNDM*, showed the highest dissemination in our sample set with 18.5% and 15.5% of the analyzed samples being positive, respectively.

Dissecting the ARG-detection by sites allowed us to identify an accumulation over time of the ARGs specifically on the floor. While the amount of total ARG counts (across the 9 tested rooms) did not increase over the 30-week span in neither the sink nor the doorhandle samples, a steady increase (up to 20 total ARG counts in week 30) was observed for the floor samples over time (Fig. 6B). Breaking it down to the singular ARGs, the most significant increase was observed for *blaVIM*, which was absent in the pre-opening week and disseminated to all floor samples in week 16 after patient occupancy. Interestingly, *blaVIM* was not detected in any of the doorhandle or sink samples. Another gene, *blaNDM*, was also highly detected on the floor (with 67% positive samples in week 16). Other genes such as *blaCMY*, *blaSHV*, and *mecA* showed highest dissemination on the floor in week 30, with 22%, 11%, and 44% of the samples being positive, respectively. The latter gene, *mecA*, was also found to be present in a few doorhandle samples and was constantly detected in the sink with 11–22% positive samples starting from week 7 (Fig. 6C).

When analyzing the presence of resistance determinants in the patient samples (rectal and nasal swabs), we detected the same 6 genes found in the environmental samples (Suppl. Fig. S12). However, the dissemination of ARGs was not as pronounced as the one observed on the floor samples. Moreover, analysis of the room-specific detection of particular ARGs in both environmental and/or patient samples showed no direct correlation between the patient-environment pairs over each week (*p* > 0.1, Chi-square test; see Suppl. Table S5).

## Discussion

This study dissects the colonization dynamics of different surfaces of the hospital environment after first patient occupancy. Such an early colonization process is not a random procedure in which cells arbitrarily attach and start growing. Instead, it is a complex process involving attachment events, movement, and bacterial interactions to yield a non-random spatial organization [56]. The maturation of the community structure will largely depend on the competition–colonization tradeoff between its members [57] and the external stress conditions [56]. Thus, the kinetic of such processes is highly variable. So, for example, a recent study addressing the colonization process of an urban wastewater treatment plant reported compositional changes over a few months until reaching a stable sewage microbiome [58]. Temporal dynamics for the bacterial colonization of plastic surfaces are even shorter, and have been reported in terms of weeks [59, 60]. The longitudinal results of our study on the colonization of hospital surfaces also point toward very short time frames for the establishment of a site-specific microbiota. In only 5–7 weeks, we observed stable microbial communities at all tested locations, which would not significantly change in their composition further on, as assessed by ANOSIM analyses on the beta-diversity over time (Fig. 2E). Moreover, in this short time frame, we observed a steady increase of the bacterial biomass and its diversity. These are two characteristics which have also been described for well-known colonization processes, such as the gut microbiome development in infants [61, 62]. Toward the end of the time series, we observed a slight decrease of the bacterial biomass in each of the analyzed sites from the hospital environment (Fig. 1A). We hypothesize that this general reduction of bacterial load might be dependent, at least in part, on seasonal fluctuations of physical factors that have an effect on the entire patient room. Air temperature and relative humidity have been reported to correlate with indoor microbial community richness in different buildings and environments [1, 16, 63, 64]. Moreover, in the clinical setting, a number of studies have already reported seasonal and temperature-associated increases of bacterial bloodstream infections [65, 66] and surgical site infections [67] in hospitalized patients. In our study, air temperature and relative humidity were recorded in all rooms at the time of sampling. Multiple regression models suggest a significant association between these two physical factors and the bacterial load measured in the room environment (Suppl. Fig. S2). These findings underscore the importance of thoughtful architectural design of new hospital buildings, with a focus on temperature and ventilation control [1, 16].

The fast maturation process of the microbial ecosystems in the hospital environment as observed by its diversity metrics was further supported by the results obtained by network analyses of their community interactions. The overall connectivity of the communities was rather low, as reflected by the clustering coefficients for all sites. These observations are in agreement with the results of a recent study investigating the environmental microbiome in different medical units in Brazil [13]. However, in our study, we observed relatively high modularity indices for all three room sites. While these values did not change over time for doorhandle and sink samples throughout the whole study, they clearly increased on the floor. In the pre-opening week, the microbial community of the floor showed modularity values of 0.215. These values almost doubled after stabilization of the site-specific community (*M* (week30) = 0.411). According to Newman (2006), values > 0.4 are indicative of a modular structure in the networks [43]. Such modularity values are positively associated with network stability and an improved resilience of the microbial communities to environmental stress factors [68]. Interestingly, the highest rise of the modularity index for the floor samples was already observed after the first week of patient occupancy (from 0.215 (w0) to 0.375 (w1)), suggesting an early onset of the modular structuring of the community and thus, of its stability. In the particular case of the floor, the fast increase of modular structures and the stabilization of its microbial composition is remarkable, as it represents the continuation of the outside environment, where a high bacterial exchange and shoe-to-floor carryover might be assumed [69].

In our study, we observe a site-specific organization of the communities, which are more divergent between each other as the community structures develop over time. Grinberg et al. (2019) suggested that such early colonization processes are driven by self-organizing mechanisms, such as preferential attachments. The authors described this process as a stochastic growth, in which individuals join existing groups and aggregate in a system in a non-random way [56]. In our system, the patient occupancy is the main driving input of new bacterial cells, which will then show its preferential attachment to different aggregates in each niche. From the patient microbiota, the skin microbiome (hand and elbow) showed the highest impact on the hospital environment, which is in agreement with previous findings [11]. Indirectly, the human action also introduces severe disturbances and landscape changes to the system in a site-specific manner. Besides daily washing/disinfection procedures, these disturbances also include the alternating wet-dry cycles of the sink. Grinberg et al. (2019) demonstrated that periodic stress, and wet-dry cycles in particular, lead to lower community fitness [56]. This might explain, at least in part, that the structure dynamics are most biased in the sink samples, as measured by the lower modularity of its network (*M* = 0.330 week 30) or the increased fluctuation observed in the beta-diversity analyses over time.

From a taxonomic point of view, the most abundant species identified in the sink was *Veillonella rogosae*, which showed a significant increase of its relative abundance over time. This species is known as one of the early colonizers in oral biofilm formation [70, 71]. In this context, *V*. *rogosae* has been reported to coaggregate with *Streptococcus* spp. and cooperate in the early stages of biofilm formation of the oral cavity [70]. Accordingly, we identified two *Streptococcus* entries among the 5 top abundant species in the sink samples, one of them being an “oral clone” (Fig. 4A). Indeed, *V*. *rogosae* and the *Streptococcus* spp. account for 32.3% of all sink species in week 30. Moreover, a significant interaction between the oral *Streptococcus* sp. and *V*. *rogosae* was identified in the network analysis of week 30 (*p* < 0.05), but not in the pre-opening week. These results might point toward the dental hygiene measures of the patients as one of the main contributors to the bacterial colonization process in the sink. This hypothesis is in agreement with previous studies reporting a dominant presence of *Veillonellaceae* in bathroom sink drainage pipes [72] as opposed to other periodically wet household surfaces, such as kitchen sinks or bathroom showers [73]. Furthermore, *Veillonella* and *Streptococcus* species were recently identified as the main bacterial taxa found on used toothbrushes by metagenomics approaches [74], which is consistent with the bacterial community structure found in our sink samples. On the floor, we identified *Acinetobacter* as the most abundant genera, showing a significant increase over time after patient occupancy. The high dominance of this single taxa contributes to an unexpected low alpha-diversity metric on the floor when compared to the other two sites. However, the observed dominance of *Acinetobacter* species on floor samples is in agreement with the findings of other studies addressing the floor microbiota of public buildings [11, 75]. While taxonomic analyses did not permit for species assignment, targeted sequence search allowed us to discard the presence (< 0.1%) of the pathogenic bacteria *Acinetobacter baumanii* sequences in our sample set. On the other side, we identified two potential pathogens (*E*. *coli* and *S*. *aureus*) as the most abundant species on the doorhandle samples. However, we did not detect a significant increase of these species over time.

From a scientific point of view, monitoring of bacterial communities in the hospital environment would not only allow for a better understanding of the growth dynamics of potential pathogens, but also to characterize the dissemination of antibiotic resistance determinants. A recent study by Gupta et al. (2019) showed an increased incidence of ARGs on floor surfaces of hospitals when compared with other building types [76]. The authors investigated the presence of three ARGs in their study, and were the first to report the presence of *blaKPC*, a mobile beta-lactamase coding ARG, on the floor surface of a hospital. In our study, we explored the presence of 12 different ARGs, most of them coding for different beta-lactamases. To our knowledge, this is the first study addressing such an amount of ARGs in the hospital environment. Six of the ARGs were detected in the room sites analyzed: *blaVIM*, *blaNDM*, *blaCMY*, *blaSHV*, *qnrB*, and *mecA*. While ARG detection on doorhandle and sink was rather random over time, we detected a site-specific increase of ARGs after patient occupancy on the floor surface. The most significant increase was observed for *blaVIM*, which was absent in the pre-opening week and steadily increased its dissemination until its detection on the floors of all 9 rooms in week 16 after patient occupancy. Plasmids with this carbapenemase-coding gene have been often detected in clinical isolates from diverse *Acinetobacter* and *Pseudomonas* species [77–79]. Interestingly, these two genera are among the 5 most abundant taxa on the floor in week 30, accounting for a combined 55.4% of the relative abundance in that week. Since the distribution of *Acinetobacter* spp. and *Pseudomonas* spp. is also highly significant between the analyzed room sites (see Table 1 for relative distribution values), it might be reasonable to assume that these species might harbor the bulk of the resistance determinants detected on the floor surface in this study. The other 2 ARGs with high incidence and increasing detection on the floor were *blaNDM* and *mecA*. In the clinical setting, these ARGs are typically isolated from *E*. *coli* and *S*. *aureus*, respectively [80, 81]. These two species ranged as top 14 and top 3 most abundant species on the floor in the last sampling week. However, the data does not allow for direct pathogen-ARG association. We can also not assume that all ARGs originate from live bacteria at the time of sampling. Further studies are needed to investigate whether mobile ARGs might bear the potential to be integrated by human pathogenic bacteria. Transformation processes and horizontal gene transfer between bacteria have been described as potential mechanisms in this context [82–84]. Moreover, ARG acquisition by natural transformation has been shown to be triggered by disinfection measures [85]. Some studies have pointed toward an underappreciated potential of the floor surfaces as source for health care-associated pathogens or ARGs [63–65]. However, epidemiological studies suggest an inconsequential contribution of microorganisms from inanimate surfaces to the incidence of HAIs [86–88]. In line with this, we did not detect any nosocomial infection with either methicillin-resistant *S*. *aureus*, extended-spectrum beta-lactamase-producing enterobacterales, or carbapenem-resistant enterobacterales among the patients enrolled in our study. Further environmental studies with an additional epidemiological focus are needed to investigate the effect strength of the floor as potential transmission source.

Our study has some limitations, which mainly include the inability to assign detected ARGs to specific bacteria, the inability to discriminate between live and death bacteria, and the limited amount of ARGs that were tested. In addition, only one ward was analyzed in this study, although on a very comprehensive sampling scheme. Further studies involving different wards and more healthcare centers should validate the results observed in this work. Furthermore, the integration of metagenomics strategies will allow the identification of particular antibiotic-resistant strains in each of the room sites. Seasonal factors could be only partially investigated in this study. More time series over a longer period of time are needed to investigate their effect on compositional aspects and not only on biomass. In addition, bacterial community structure might be different in older buildings, and on different surface materials. Further comparative analysis between hospitals and cleaning regimes might add valuable information at the epidemiological and the microbiological level for the refurbishment or renovation of older health care buildings.

## Conclusions

In conclusion, this study describes the early bacterial colonization dynamics of the hospital environment upon initial patient occupancy. We report a site-specific development of the microbial populations on doorhandle, sink, and floor surfaces, leading to stable community structures in only a few weeks after patient occupancy. This colonization is characterized by an initial increase in the bacterial load and its diversity. In the particular case of the floor surface, the colonization process is associated with a significant rise of antibiotic-resistance determinants over time. This data contribute to a novel understanding of the environmental microbiota in the hospital setting.

## Supplementary Information

**Additional file 1: Suppl. Figure S1**. Workflow schematic of the longitudinal study. Shown is the map depicting the 9 rooms of the neurological ward in which 1547 samples were collected including 3 environmental sites and 4 patient sites over a time course of 31 weeks (Pre-opening week + 30 weeks after initial patient occupancy). The metagenetic pipelines included *16S rRNA* quantification and sequencing steps, as well as Taq-Man assays for ARG-detection. **Suppl. Figure S2**. Correlation between physical parameters and bacterial load. Shown are different multiple regression models which beside location include either the temperature, the humidity or both factors as potential contributors to the bacterial load as measured by *16S rRNA* copies (qPCR). The highest r2 value and the best model fit was achieved when combining both temperature and humidity in the regression model. (C/E: copies per extraction). **Suppl. Figure S3**. Alpha- and beta-diversity metrics used for systematic subsampling in week-blocks. A) Shown are the overall alpha-diversity values of the collapsed environmental microbiome of each of the 31 weeks. Significant increase was observed after the first initial 4-week block. B) PCA of the collapsed environmental microbiome data for all 31 week (each week represented by a single dot). The coloring scheme is based on the distance pattern between consecutive weeks. The second block (8 weeks, green) was further divided in two halves to allow a more detailed analysis of the critical period of community stabilization, which was shown to occur between weeks 5 and 7 after patient occupancy. **Suppl. Figure S4**. Alpha-diversity metrics of the different room sites over time. Shown are the Shannon indices (Median and IQR 25-75%) of floor, doorhandle and sink samples across different week blocks. **Suppl. Figure S5**. Distance comparison between sites in the pre-opening and last sampling weeks. Shown are the weighted UniFrac distances between site pairs as measured in week 0 and in week 30 (Mean±SEM; *p<0.05; **p<0.01). **Suppl. Figure S6**. No impact of spatial room distribution on microbiome patterns. A) Principal Coordinate plots show the distribution of the microbiome data based on the distribution of the rooms across the ward. Shown are the p-values obtained by PERMANOVA test. B) The floor plan shows two clusters of contiguous rooms among the sampled sites, which are located on opposing sides of the neurology ward. **Suppl. Figure S7**. Patient microbiome patterns in this study. A) Collapsed taxonomic summary of the nasal and rectal swabs collected from the patients during the first 30 weeks of occupancy. B) Heatmaps showing the amount of shared taxa (at family level) between the core microbiomes of the environmental- and the patient-samples for different week-blocks. C) Taxonomic summary of the hand and elbow samples collected during the final 6 weeks of the time series. **Suppl. Figure S8**. Correlation plots between alpha-diversity metrics and external factors (temperature and humidity). Shown are the correlations between the temperature (°C) or the humidity (%) and the alpha-diversity of the three environmental sites. **Suppl. Figure S9**. PCoA of the beta-diversity depicting the distances between environmental and patient samples. Shown are the weighted UniFrac distances between the environmental cluster (grey) to each of the patient sites individually (colored). **Suppl. Figure S10**. Dynamics of the bacterial community structure over time. A) Relative abundance changes over time of the most abundant families. Bubble size represents the proportional abundance across different week blocks. B) Relative abundance dynamics of the top significantly changed taxa (family level) across the different week blocks. **Suppl. Figure S11**. Pathogen colonization of the hospital environment. A) Bar-chart depicting the incidence of pathogenic bacteria in the analyzed environmental samples. Shown are the number of samples positive for *C. difficile* or any of the 10 most frequent pathogens isolated from Charité-patients during the sampling period. B) Relative abundance of the detected pathogen sequences over time. Shown are the patterns across different week blocks. **Suppl. Figure S12**. ARG detection in the patient samples (rectum and nare) over selected weeks. Chart depicts the percentage of positive samples in each week for each of the ARGs. **Suppl. Table S1**. Basic epidemiological parameters of the patients sampled in this study. **Suppl. Table S2**. Library PCR-constructs. Shown are the sequences of the Fw- and Rv-primers used for library construction. **Suppl. Table S3**. List of nodes from the network analysis, indicating their pathogenicity status and the supporting reference source. When the taxa was not listed in any of the pathogen databases (KEGG Pathogens database (https://www.kegg.jp/kegg/genome/pathogen.html); ISID: Database of The International Society for Infectious Diseases (ISID) (https://isid.org/)), literature research was performed with the species name. Reports on opportunistic pathogenicity were referenced where possible. In all other cases, the node was defined as non-pathogen/unclassified. **Suppl. Table S4**. List of all ARGs addressed in this study, indicating the sequences of the designed primers and probes used for their detection via Taq-Man assay. In all cases, primers were designed to cover maximum number of known gene variants. **Suppl. Table S5**. Correlation tests between the occurrence of specific ARGs in Patient AND Environment Sites in a particular week. Shown are the p-values obtained for each correlation (Chi-Square test).

## Data Availability

The datasets generated for this study are available in the SRA database under the accession number: PRJNA672813 [https://www.ncbi.nlm.nih.gov/sra/ PRJNA672813].
