# Supplementary material for: Bacterial colonization dynamics and antibiotic resistance gene dissemination in the hospital environment after first patient occupancy: a longitudinal metagenetic study
Source: Microbiome. 2021 Aug 11;9:169. doi: 10.1186/s40168-021-01109-7 (PMC8359561; doi:10.1186/s40168-021-01109-7)
Supplement: Supplementary file 2 — Additional file 1: Suppl. Figure S1. Workflow schematic of the longitudinal study. Shown is the map depicting the 9 rooms of the neurological ward in which 1547 samples were collected including 3 environmental sites and 4 patient sites over a time course of 31 weeks (Pre-opening week + 30 weeks after initial patient occupancy). The metagenetic pipelines included 16S rRNA quantification and sequencing steps, as well as Taq-Man assays for ARG-detection. Suppl. Figure S2. Correlation between physical parameters and bacterial load. Shown are different multiple regression models which beside location include either the temperature, the humidity or both factors as potential contributors to the bacterial load as measured by 16S rRNA copies (qPCR). The highest r2 value and the best model fit was achieved when combining both temperature and humidity in the regression model. (C/E: copies per extraction). Suppl. Figure S3. Alpha- and beta-diversity metrics used for systematic subsampling in week-blocks. A) Shown are the overall alpha-diversity values of the collapsed environmental microbiome of each of the 31 weeks. Significant increase was observed after the first initial 4-week block. B) PCA of the collapsed environmental microbiome data for all 31 week (each week represented by a single dot). The coloring scheme is based on the distance pattern between consecutive weeks. The second block (8 weeks, green) was further divided in two halves to allow a more detailed analysis of the critical period of community stabilization, which was shown to occur between weeks 5 and 7 after patient occupancy. Suppl. Figure S4. Alpha-diversity metrics of the different room sites over time. Shown are the Shannon indices (Median and IQR 25-75%) of floor, doorhandle and sink samples across different week blocks. Suppl. Figure S5. Distance comparison between sites in the pre-opening and last sampling weeks. Shown are the weighted UniFrac distances between site pairs as measured in week 0 and in w [file 40168_2021_1109_MOESM2_ESM.pdf]

SUPPLEMENTARY MATERIAL

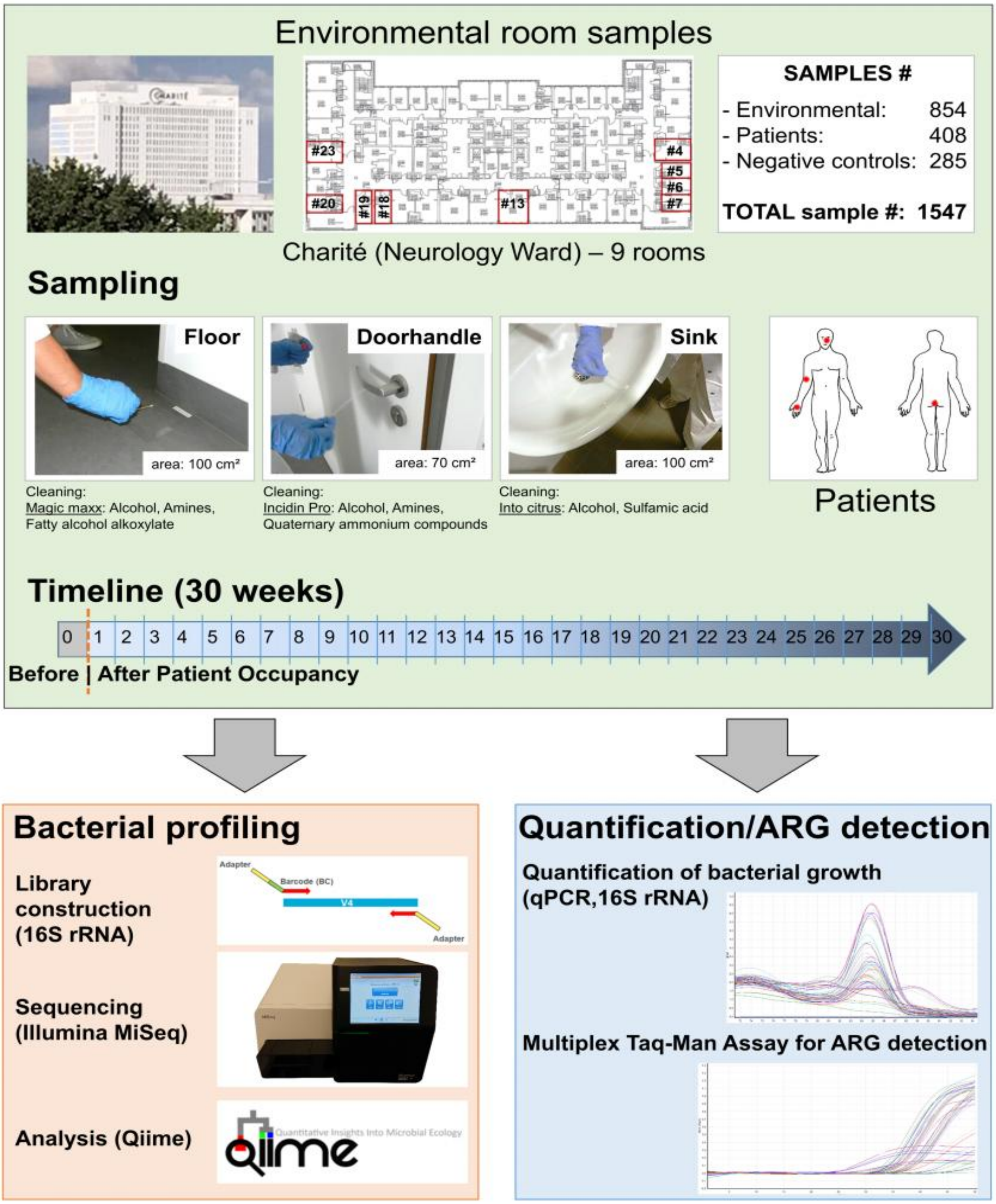

Suppl. Figure S1.- Workflow schematic of the longitudinal study. Shown is the map depicting the 9 rooms of the neurological ward in which 1547 samples were collected including 3 environmental sites and 4 patient sites over a time course of 31 weeks (Pre-opening week + 30 weeks after initial patient occupancy). The metagenetic pipelines included *16S rRNA* quantification and sequencing steps, as well as Taq-Man assays for ARG-detection.

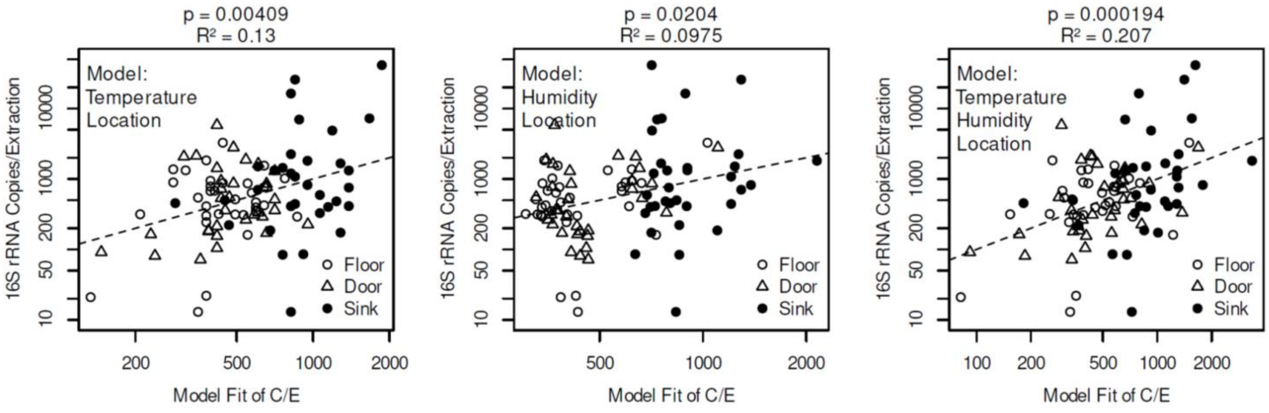

Suppl. Figure S2.- Correlation between physical parameters and bacterial load. Shown are different multiple regression models which beside location include either the temperature, the humidity or both factors as potential contributors to the bacterial load as measured by *16S rRNA* copies (qPCR). The highest r2 value and the best model fit was achieved when combining both temperature and humidity in the regression model. (C/E: copies per extraction).

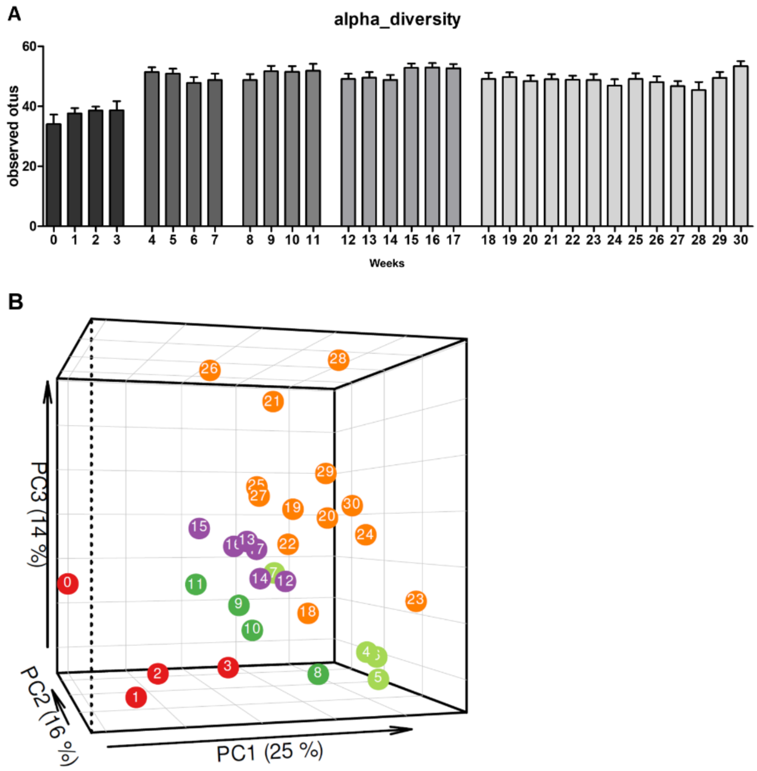

Suppl. Figure S3.- Alpha- and beta-diversity metrics used for systematic subsampling in week-blocks. A) Shown are the overall alpha-diversity values of the collapsed environmental microbiome of each of the 31 weeks. Significant increase was observed after the first initial 4-week block. B) PCA of the collapsed environmental microbiome data for all 31 week (each week represented by a single dot). The coloring scheme is based on the distance pattern between consecutive weeks. The second block (8 weeks, green) was further divided in two halves to allow a more detailed analysis of the critical period of community stabilization, which was shown to occur between weeks 5 and 7 after patient occupancy.

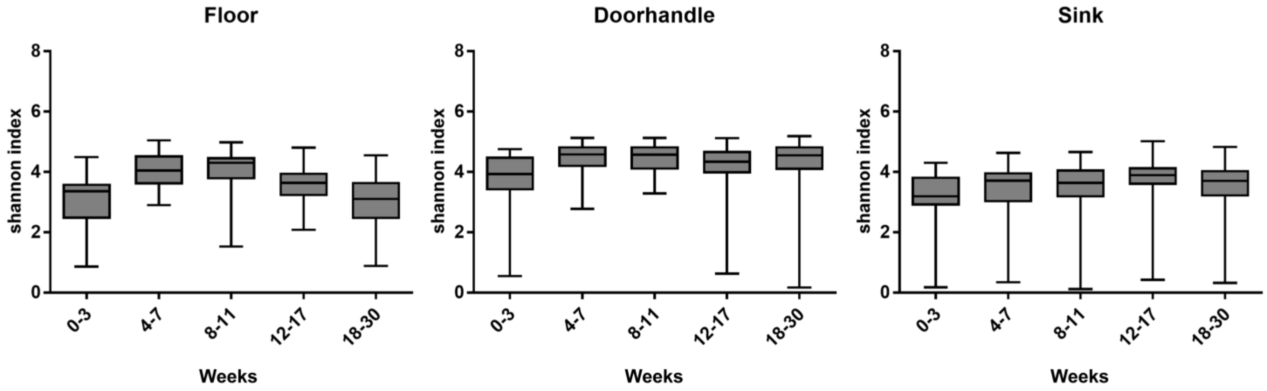

Suppl. Figure S4.- Alpha-diversity metrics of the different room sites over time. Shown are the Shannon indices (Median and IQR 25-75%) of floor, doorhandle and sink samples across different week blocks.

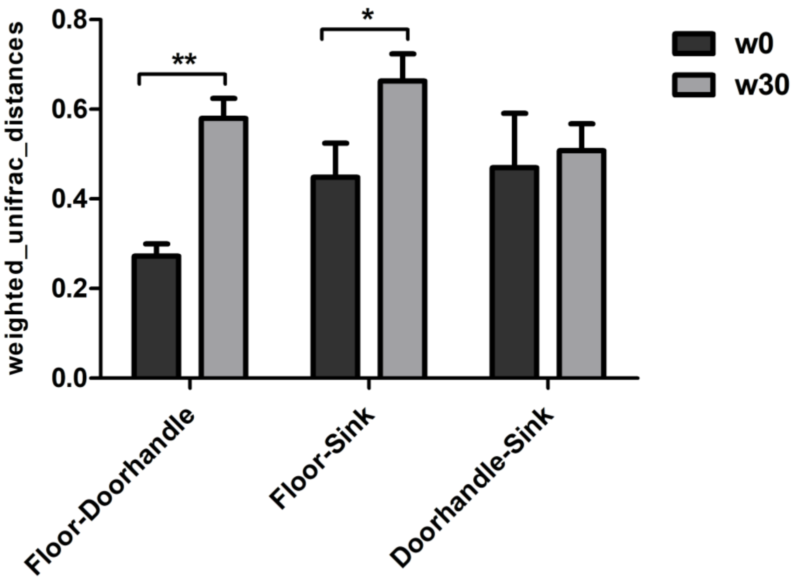

Suppl. Figure S5.- Distance comparison between sites in the pre-opening and last sampling weeks. Shown are the weighted UniFrac distances between site pairs as measured in week 0 and in week 30 (Mean±SEM; *p<0.05; **p<0.01).

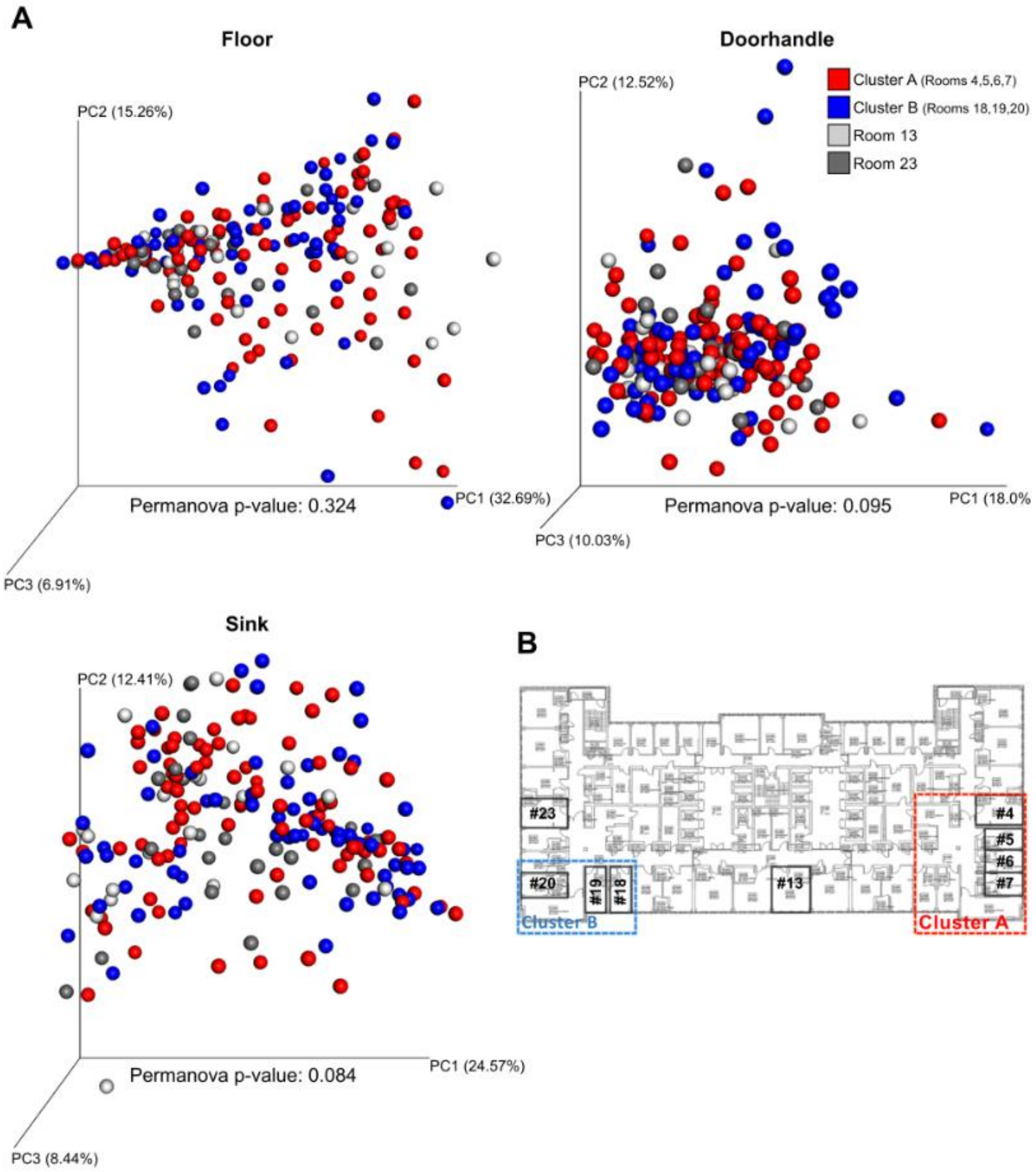

Suppl. Figure S6.- No impact of spatial room distribution on microbiome patterns. A) Principal Coordinate plots show the distribution of the microbiome data based on the distribution of the rooms across the ward. Shown are the p-values obtained by permanova test. B) The floor plan shows two clusters of contiguous rooms among the sampled sites, which are located on opposing sides of the neurology ward.

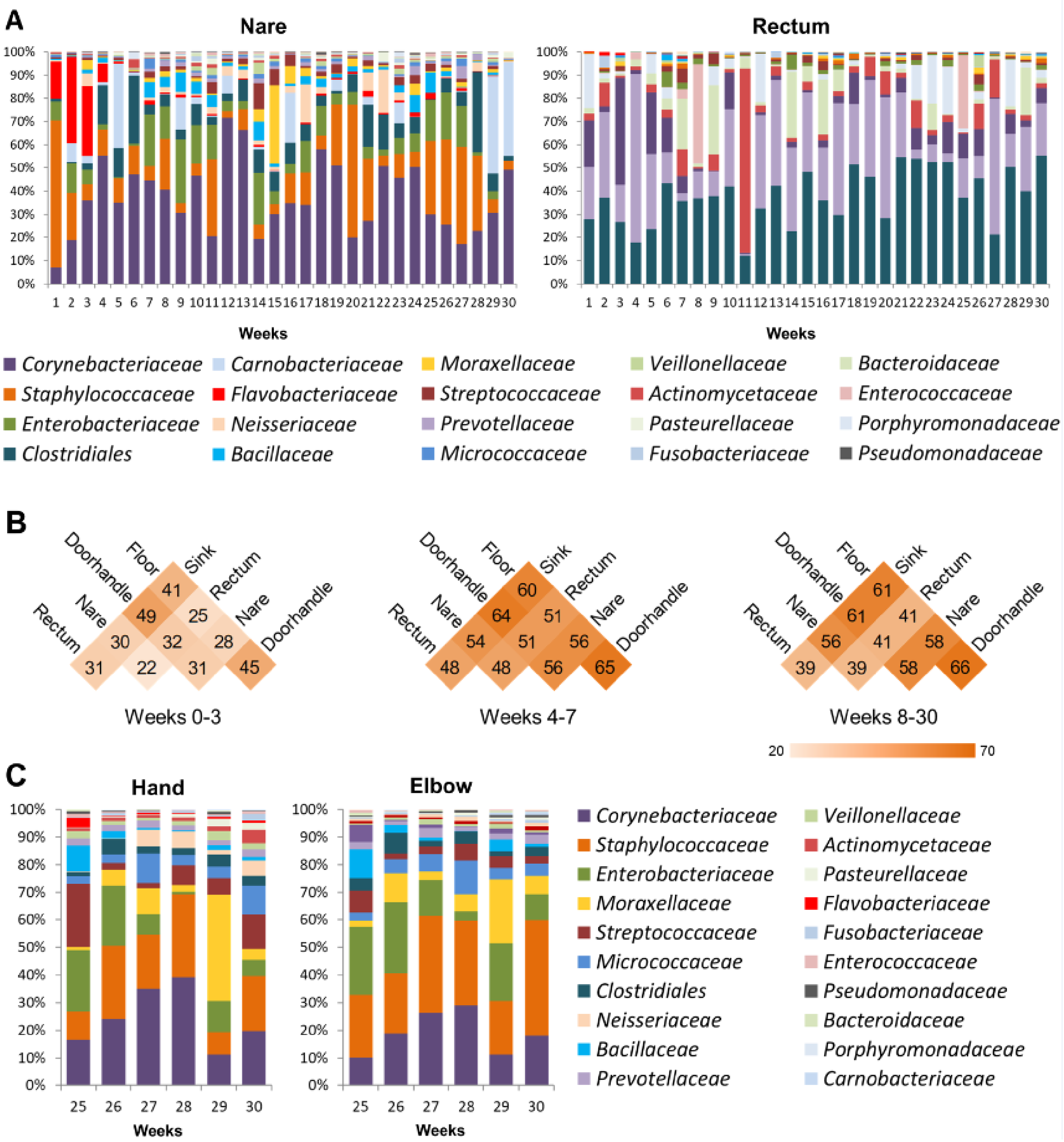

Suppl. Figure S7.- Patient microbiome patterns in this study. A) Collapsed taxonomic summary of the nasal and rectal swabs collected from the patients during the first 30 weeks of occupancy. B) Heatmaps showing the amount of shared taxa (at family level) between the core microbiomes of the environmental- and the patient-samples for different week-blocks. C) Taxonomic summary of the hand and elbow samples collected during the final 6 weeks of the time series.

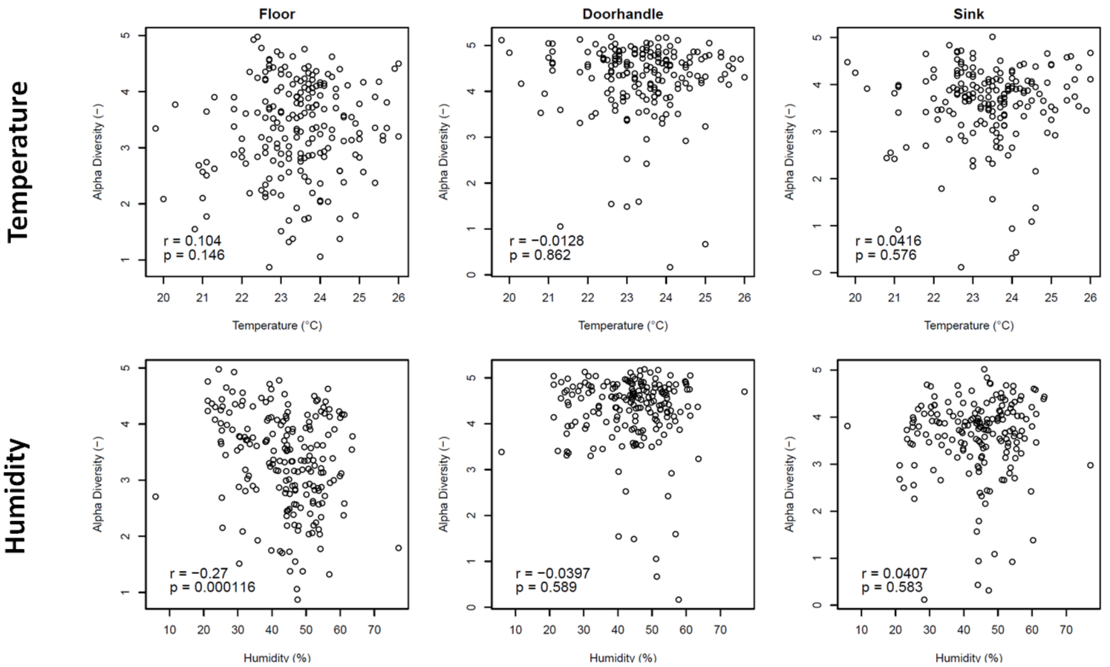

Suppl. Figure S8.- Correlation plots between alpha-diversity metrics and external factors (temperature and humidity). Shown are the correlations between the temperature (°C) or the humidity (%) and the alpha-diversity of the three environmental sites.

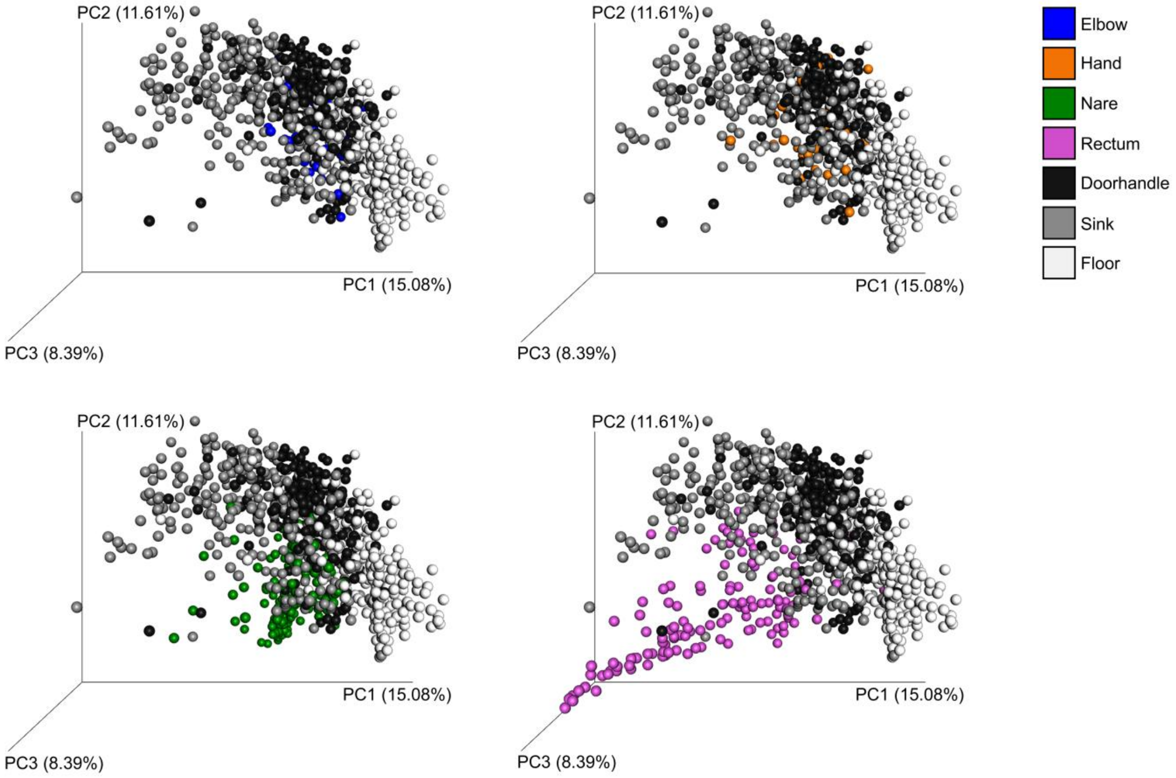

Suppl. Figure S9.- PCoA of the beta-diversity depicting the distances between environmental and patient samples. Shown are the weighted UniFrac distances between the environmental cluster (grey) to each of the patient sites individually (colored).

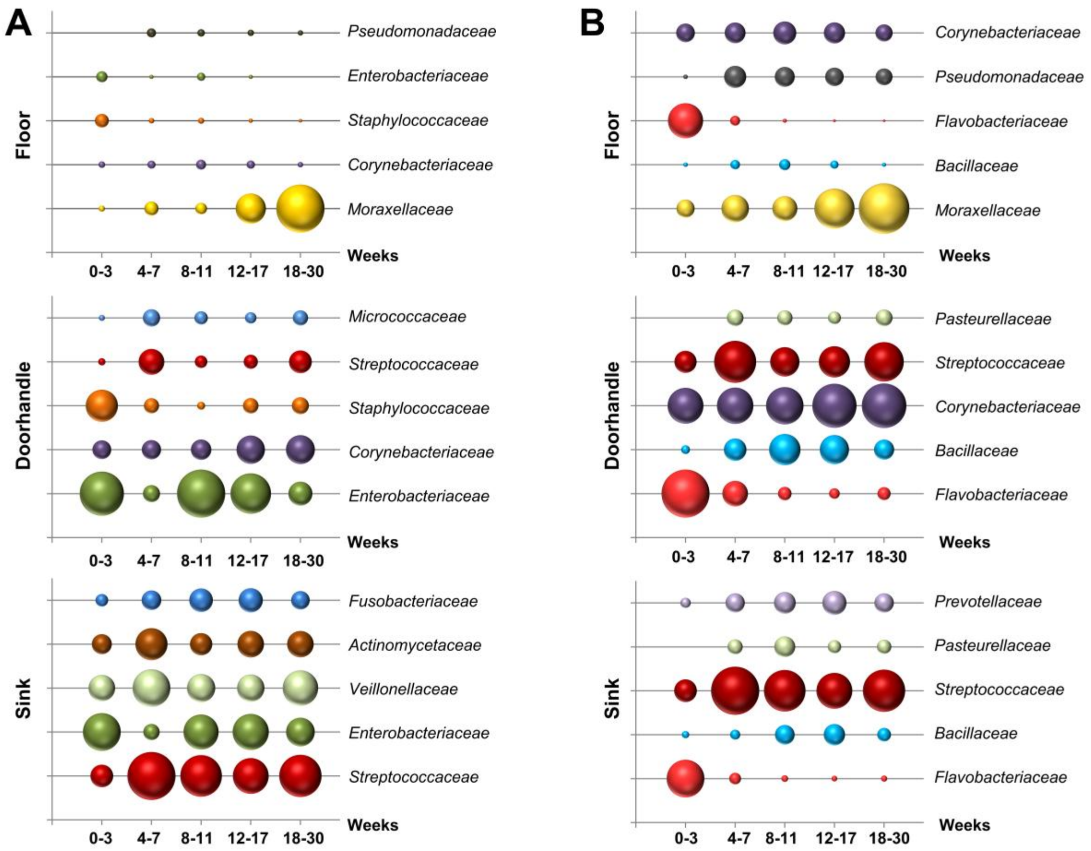

Suppl. Figure S10.- Dynamics of the bacterial community structure over time. A) Relative abundance changes over time of the most abundant families. Bubble size represents the proportional abundance across different week blocks. B) Relative abundance dynamics of the top significantly changed taxa (family level) across the different week blocks.

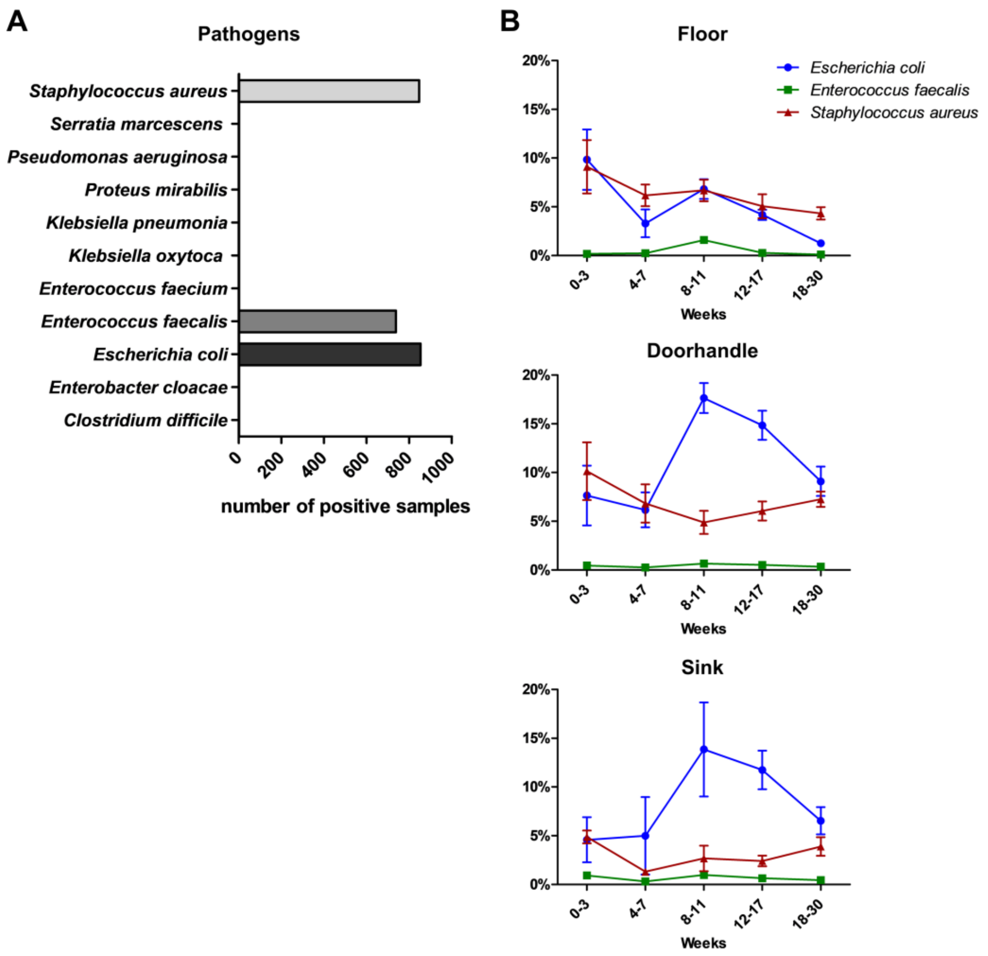

Suppl. Figure S11.- Pathogen colonization of the hospital environment. A) Bar-chart depicting the incidence of pathogenic bacteria in the analyzed environmental samples. Shown are the number of samples positive for *C. difficile* or any of the 10 most frequent pathogens isolated from Charité-patients during the sampling period. B) Relative abundance of the detected pathogen sequences over time. Shown are the patterns across different week blocks.

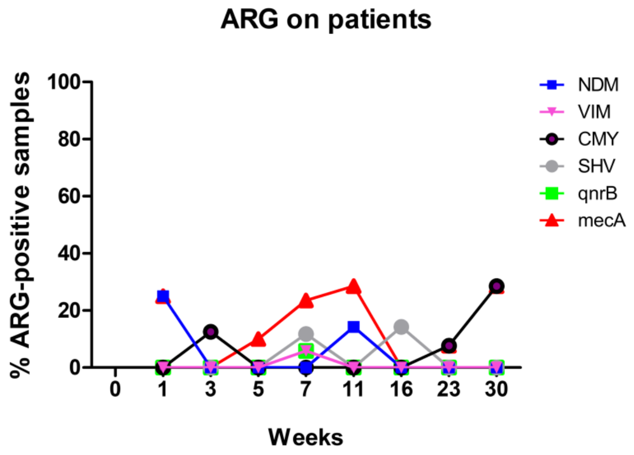

Suppl. Figure S12.- ARG detection in the patient samples (rectum and nare) over selected weeks. Chart depicts the percentage of positive samples in each week for each of the ARGs.

Suppl. Table S1.- Basic epidemiological parameters of the patients sampled in this study.

Parameter	Total (n=152)
Length of stay overall (Median, IQR)	5 (3-7)
Length of stay until sampling (Median, IQR)	2 (0-4)
Age (Median, IQR)	55 (43-71)
Charlson comorbidity score (Median, IQR)	3 (1-6)
Male (%, number)	51.3% (78)
Myocardial infarction (%, number)	0.6% (1)
Congestive heart failure (%, number)	1.9% (3)
Peripheral vascular disease (%, number)	6.5% (10)
Cerebrovascular disease (%, number)	17.1% (26)
Dementia (%, number)	4.6% (7)
Chronic lung disease (%, number)	5.2% (8)
Connective tissue disease (%, number)	7.2% (11)
Pepctic ulcer (%, number)	2.6% (4)
Mild liver disease (%, number)	2.6% (4)
Diabtetes without chronic complication (%, number)	13.8% (21)
Diabetes with chronic complication (%, number)	0.6% (1)
Renal disease (%, number)	37.5% (57)
Malign tumor (%, number)	5.2% (8)
Moderate to severe liver disease (%, number)	none
Metastatic cancer (%, number)	none
AIDS/HIV (%, number)	1.3% (2)
Hemiplegia (%, number)	26.3% (40)
Leukemia (%, number)	none
Lymphoma (%, number)	none
Infection per ICD code (A49.-) (%, number)	27.6% (42)

Suppl. Table S2.- Library PCR-constructs. Shown are the sequences of the Fw- and Rv-primers used for library construction.

Forward construct	Construct part	Sequence
	5´Illumina adapter	AATGATACGGCGACCACCGAGATCTACACGCT
	Golay barcode	NNNNNNNNNNNN
	Forward primer pad	TATGGTAATT
	Forward linker	GT
	Forward primer (515Fw)	GTGYCAGCMGCCGCGGTAA
**Reverse construct**	**Construct part**	**Sequence**
	3´Illumina adapter	CAAGCAGAAGACGGCATACGAGAT
	Reverse primer pad	AGTCAGCCAG
	Reverse linker	CC
	Reverse primer (806Rv)	GGACTACNVGGGTWTCTAAT

Suppl. Table S3.- List of nodes from the network analysis, indicating their pathogenicity status and the supporting reference source. When the taxa was not listed in any of the pathogen databases (KEGG Pathogens database (https://www.kegg.jp/kegg/genome/pathogen.html); ISID: Database of The International Society for Infectious Diseases (ISID) (https://isid.org/)), literature research was performed with the species name. Reports on opportunistic pathogenicity were referenced where possible. In all other cases, the node was defined as non-pathogen/unclassified.

		Source		
**OTUs**	**Description**	**KEGG**	**ISID**	**References**
Acinetobacter sp.	non-pathogen/unclassified	no	no	
Anaerococcus vaginalis	opportunistic pathogen	no	no	DOI: 0.1099/00207713-51-4-1521
Anaerococcus sp.	non-pathogen/unclassified	no	no	
Corynebacterium glyciniphilum	non-pathogen/unclassified	no	no	
Corynebacterium kroppenstedtii	opportunistic pathogen	no	no	DOI:10.1016/j.ijid.2016.04.023
Corynebacterium pseudodiphtheriticum	opportunistic pathogen	no	no	DOI:10.1093/clinids/20.1.37
Corynebacterium segmentosum	non-pathogen/unclassified	no	no	
Corynebacterium testudinoris	non-pathogen/unclassified	no	no	
Corynebacterium ureicelerivorans	opportunistic pathogen	no	no	DOI: 10.1007/s10096-008-0677-1.
Dolosigranulum pigrum	opportunistic pathogen	no	no	DOI: 10.1128/JCM.01373-07
Enterococcus faecalis	pathogen	yes	yes	
Escherichia coli	pathogen	yes	yes	
Fenollaria timonensis	non-pathogen/unclassified	no	no	
Finegoldia magna	opportunistic pathogen	no	no	DOI:10.1016/j.mehy.2012.04.013
Flavobacterium succinicans	non-pathogen/unclassified	no	no	
Fusobacterium nucleatum	pathogen	yes	no	PMID: 21220789
Lactococcus lactis subsp. lactis	opportunistic pathogen	no	no	DOI: 10.1097/MD.0000000000013658
Lawsonella clevelandensis	opportunistic pathogen	no	no	DOI:10.1016/j.idcr.2018.03.014
Micrococcus sp.	non-pathogen/unclassified	no	no	
Neisseria meningitidis	pathogen	yes	yes	
Pasteurella aerogenes	non-pathogen/unclassified	no	no	
Peptoniphilus sp.	non-pathogen/unclassified	no	no	
Prevotella sp.	non-pathogen/unclassified	no	no	
Prevotella bivia	non-pathogen/unclassified	no	no	
Prevotella buccalis	opportunistic pathogen	no	no	DOI:10.1371/journal.pone.0238705
Prevotella copri	opportunistic pathogen	no	no	DOI: 10.1186/s13099-019-0325-6
Pseudomonas sp.	non-pathogen/unclassified	no	no	
Rheinheimera sp.	non-pathogen/unclassified	no	no	
Rothia mucilaginosa	opportunistic pathogen	no	no	DOI:10.3109/00365548.2014.980843
Staphylococcus aureus	pathogen	yes	yes	
Streptococcus sp. oral clone	non-pathogen/unclassified	no	no	
Streptococcus thermophilus	non-pathogen/unclassified	no	no	
Streptococcus sp.	non-pathogen/unclassified	no	no	
Varibaculum sp. Marseille	non-pathogen/unclassified	no	no	
Veillonella rogosae	non-pathogen/unclassified	no	no	

Suppl. Table S4.- List of all ARGs addressed in this study, indicating the sequences of the designed primers and probes used for their detection via Taq-Man assay. In all cases, primers were designed to cover maximum number of known gene variants.

ARGs	Forward primers (5´-3´)	Reverse primers (5´-3´)	Reporters/Probes (5´-3´)	length	Dye
KPC	CTGTATCGCCGTCTAGTTCTG	AGTTTAGCGAATGGTTCCG	TGTCTTGTCTCTCATGGCCGCTGG	24	FAM
NDM	GCATTAGCCGCTGCATT	GATCGCCAAACCGTTGG	ACGATTGGCCAGCAAATGGAAACTGG	26	ROX
OXA48	TTCCCAATAGCTTGATCGC	CCATCCCACTTAAAGACTTGG	TCGATTTGGGCGTGGTTAAGGATGAAC	27	HEX
VIM	TGGCAACGTACGCATCACC	CGCAGCACCGGGATAGAA	TCTCTAGAAGGACTCTCATCGAGCGGG	27	Cya5
CMY	GGAGAAAACGCTCCAGCA	GGCCAGTTCAGCATCTCC	TCTCGCTACTGGCGTATTGGCGA	23	FAM
GES	GCCTATTGCTATGGCACGT	CTTTAGGAAAACCCGCTCGT	TGGCTGATCGGAAACCAAACGGG	23	ROX
SHV	CCGATGAACGCTTTCCCA	CGGCGAGTAGTCCACCA	CTGGCGCGGGTGGATGCC	18	Cya5
TEM	GAGCTGAATGAAGCCATACCA	CCGCCTCCATCCAGTCT	GTTGCGCAAACTATTAACTGGCGAACTACT	30	FAM
ctxM1	GGACGATGTCACTGGCTG	TCGTCTCCCAGCTGTCG	AGCGATAACGTGGCGATGAATAAGCTG	27	ROX
mcr1	GCGTTCAGCAGTCATTATGC	CGAGTAGATTGGCATGATCGG	GCATAAGCCGCTGCGTAGCTATGT	24	HEX
mecA	GATTCAGGTTACGGACAAGGT	GTGAGGTGCGTTAATATTGCC	ACCCAGTACAGATCCTTTCAATCTATAGCGC	31	HEX
qnrB	TCGACTTTCGACTGGCGA	AGCTTAACGCCTTGTAAATCAAC	CACACATTGCGATCTGACCAATTCGGA	27	Cya5

Suppl. Table S5.- Correlation tests between the occurrence of specific ARGs in Patient AND Environment Sites in a particular week. Shown are the p-values obtained for each correlation (Chi-Square test).

Correlation	X^2 p-value
Patient-Floor	1
Patient-Doorhandle	1
Patient-Sink	0.12
